# Current strategies for armoring chimeric antigen receptor T-cells to overcome barriers of the solid tumor microenvironment

**DOI:** 10.3389/fimmu.2025.1643941

**Published:** 2025-09-11

**Authors:** Dorothy D. Yang, William Macmorland, James N. Arnold

**Affiliations:** School of Cancer and Pharmaceutical Sciences, King’s College London, London, United Kingdom

**Keywords:** chimeric antigen receptor, armored, T-cells, immunotherapy, solid tumors, microenvironment, stroma, immunosuppression

## Abstract

Chimeric antigen receptor (CAR) T-cell therapy is a transformative immunotherapeutic approach, yet its application in solid tumors is hindered by the immunosuppressive tumor microenvironment (TME). The TME restricts T-cell trafficking, impairs effector functions, and promotes exhaustion through soluble factors, metabolic stress, and suppressive cell populations. Recent efforts to enhance CAR T-cell efficacy have focused on armoring strategies that ‘reprogram’ and ‘boost’ T-cell responses within the TME. These include engineered expression of dominant-negative receptors or cytokine-releasing constructs (such as IL-12 and IL-18) to reshape the local immune milieu and improve T-cell effector function, synthetic Notch receptors for inducible gene expression, and chemokine receptor knock-ins to improve tumor infiltration. Additional approaches aim to modulate intrinsic metabolic pathways to improve CAR T-cell persistence under hypoxic or nutrient-deprived conditions. Armoring strategies that recruit bystander or endogenous immune cells also activate broader anti-tumor immunity that prevents antigen escape and may induce more durable anti-tumor responses. This review highlights the molecular and cellular mechanisms by which current armoring strategies enhance CAR T-cell functions in solid tumors, offering a perspective on improving immune cell engineering for overcoming the hurdles encountered in deploying these therapies against solid cancers.

## Introduction

Chimeric antigen receptor (CAR) T-cell therapy has transformed the treatment of hematological malignancies, with seven CAR T-cell therapies approved by the US Food and Drug Administration for indications including relapsed or refractory B-cell lymphomas and multiple myeloma (1–3). This technology provides a method by which a patient’s T-cells are genetically engineered to express CARs that recognize tumor-associated antigens (TAAs). Upon antigen engagement, CARs trigger T-cell activation and cytotoxicity, leading to the targeted elimination of malignant cells (4, 5). In hematological cancers, CAR T-cells have demonstrated potent immune responses and potential for achieving long-term disease remission (2). However, CAR T-cell therapy has not yet achieved comparable clinical success in solid tumors. The immunosuppressive tumor microenvironment (TME), tumor antigen heterogeneity, and limited T-cell infiltration pose major barriers to efficacy (1, 4, 6, 7). Continued innovation in CAR design and function is therefore essential to overcome these challenges and expand the therapeutic impact of CAR T-cell therapies to solid malignancies.

The basic structure of a CAR comprises an extracellular antigen-binding domain that recognizes TAAs, a transmembrane domain, and an intracellular activation signaling domain. The extracellular domain most commonly consists of a single-chain variable fragment (scFv) that binds to the target antigen (1). The intracellular domain, derived from CD3ζ, delivers a pseudo-T-cell receptor (TCR) activation signal upon CAR engagement, initiating downstream killing pathways (4, 5).

**Figure 1:**
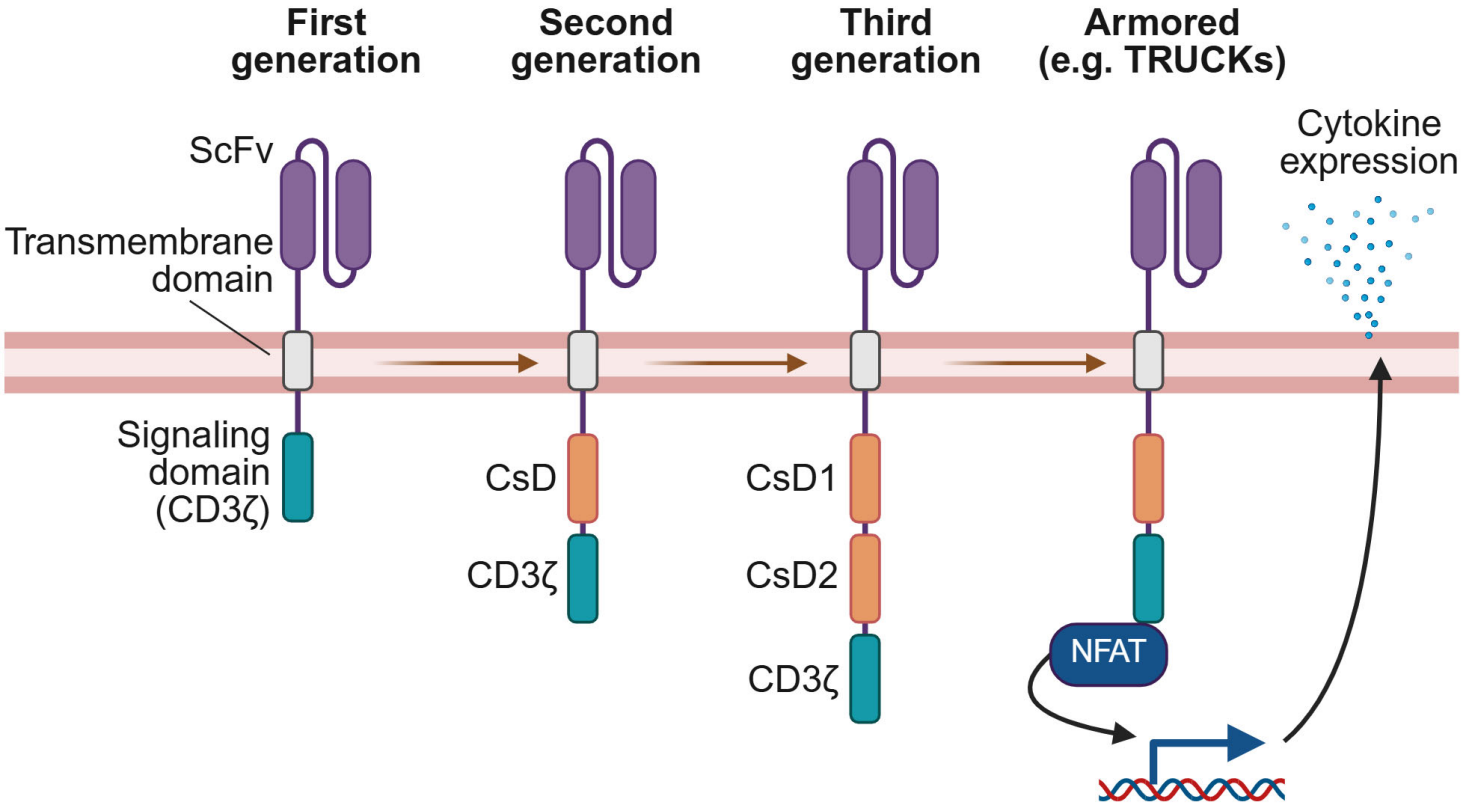
Generations of CAR technology. CARs have evolved from the original design of the first generation, with each subsequent generation incorporating new features to enhance CAR-elicited T-cell effector functions. First generation CARs comprise an extracellular domain with an antigen-specific scFv, a transmembrane domain, and an intracellular signaling domain derived from CD3ζ. Second generation CARs add a co-stimulatory domain (CsD), most commonly 4-1BB or CD28, while third generation CARs include two co-stimulatory domains. Armored CAR T-cells introduce co-expression of a biological payload that augments effector functions or persistence. For example, as illustrated here, TRUCKs are a type of armored CAR T-cell co-expressing a pro-inflammatory cytokine payload whose expression may be regulated by an NFAT-responsive promoter, linking release of the cytokine to CAR signaling. This figure was created using BioRender.com.

Innovative generations of CAR designs have been developed to enhance the functionality and clinical benefits observed from the original design (**Figure 1**). Second generation CARs include a co-stimulatory domain (CsD), consisting most commonly of either 4-1BB or CD28, which has been demonstrated to improve CAR T-cell persistence, cytokine production, and potency (4, 8). Third generation CARs combine two co-stimulatory domains for a more potent response (4, 9). Subsequent generations have focused on further augmenting effector functions or persistence, for instance by co-expressing a biological payload such as a cytokine or chemokine alongside the CAR (4, 9, 10). These innovations are the focus of this review.

**Figure 2:**
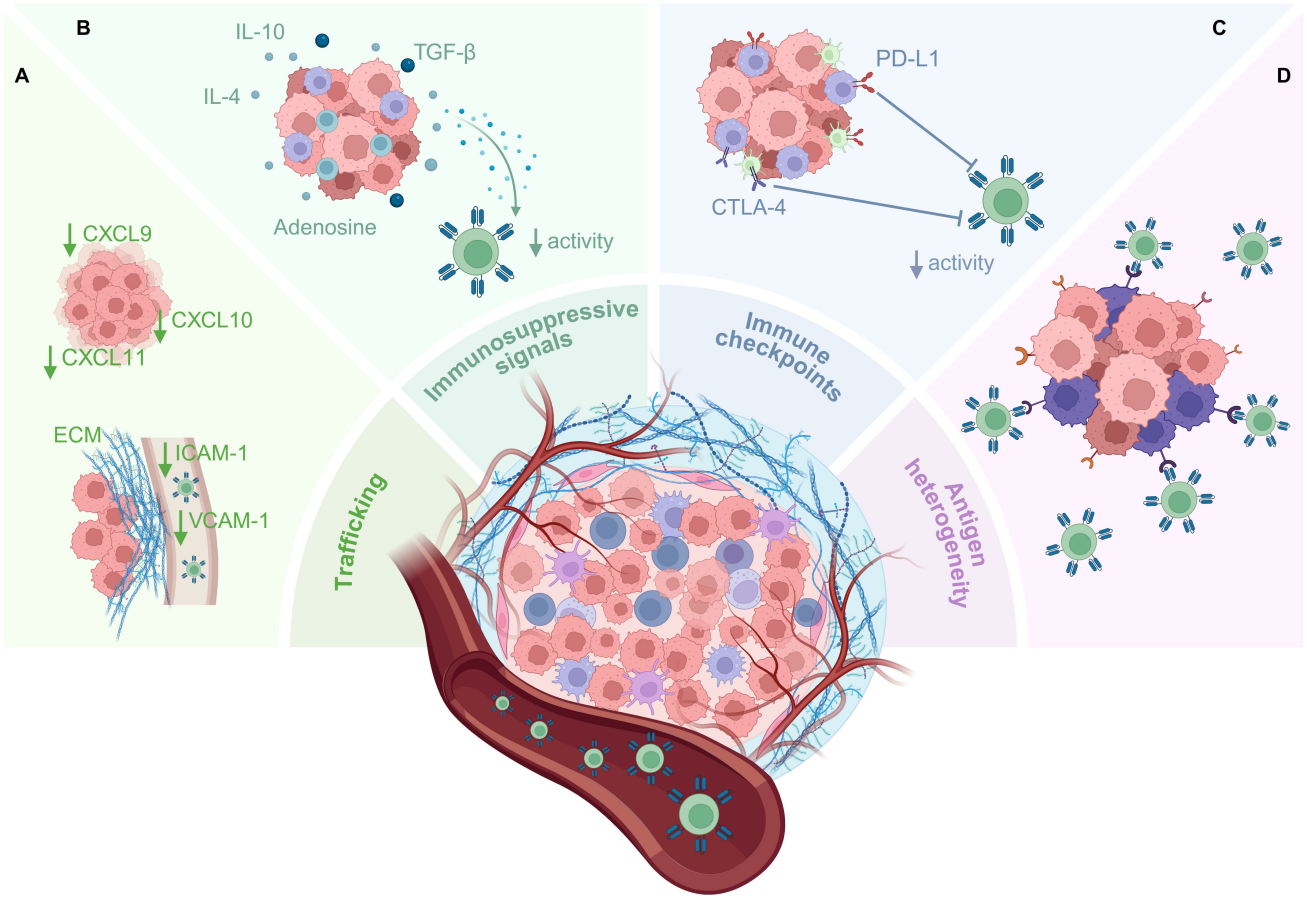
Barriers to CAR T-cell therapy in the solid TME. The solid TME poses multiple barriers to CAR T-cell infiltration and functionality which significantly impact anti-tumor efficacy. **(A)** Downregulation of chemokine ligands by the tumor can lead to inefficient trafficking of CAR T-cells to tumor sites, and downregulation of adhesion molecules such as VCAM-1 and ICAM-1 limits the migration of CAR T-cells from blood vessels into the tumor. CAR T-cells that do arrive at the tumor site are confronted by the stroma and ECM that surround solid tumors, limiting their infiltration into the tumor. **(B)** Immune and stromal cells within the TME, including CAFs, Tregs, MDSCs, and TAMs, can release immunosuppressive cytokines which reduce the function of CAR T-cells. Other immunosuppressive molecules, such as adenosine, also accumulate within the metabolically stressful milieu of the TME. **(C)** Inhibitory immune checkpoint molecules expressed by tumor and immune cells can activate immunosuppressive signaling axes within the CAR T-cells, inducing exhaustion/suppressing effector functions. **(D)** Target antigen heterogeneity allows some tumor cells to evade CAR T-cell killing, resulting in antigen escape and lack of tumor control. This figure was created using BioRender.com.

The primary distinction between solid and hematological cancers lies in the presence of a TME which can limit T-cell infiltration and functionality (**Figure 2**). Consequently, CAR T-cells face numerous obstacles that hinder successful clinical outcomes (6, 10, 11). One major challenge is the inefficient homing of T-cells to the tumor, which can result from reduced expression of chemokine ligands for effector T-cells, including CXCL9, CXCL10, and CXCL11, in the TME, impairing T-cell infiltration (12–14). Additionally, the movement of T-cells from blood vessels to the tumor site is hindered by the downregulation of extravasation mediators, including vascular cell adhesion molecule-1 (VCAM-1) and intercellular adhesion molecule-1 (ICAM-1) (15–17). T-cells that successfully cross these barriers into the TME are then confronted by a collagen-rich stromal network formed by cancer-associated fibroblasts (CAFs), which can obstruct T-cell access to tumor cells (18). CAFs, along with immune cells such as regulatory T-cells (Tregs), myeloid-derived suppressor cells (MDSCs), and tumor-associated macrophages (TAMs), contribute further to the hostile environment by releasing immunosuppressive cytokines including interleukin (IL)-4, IL-10, and transforming growth factor (TGF)-β, which reduce T-cell function (6, 19). Upregulated expression of immune checkpoint proteins, such as PD-L1 and CTLA-4, on tumor cells and immune cells within the TME also activate immune-inhibitory signaling axes which curtail CAR T-cell activity and induce functional exhaustion (6, 19). Lastly, TAA heterogeneity, arising from genomic instability and clonal evolution, can represent a mechanism of antigen escape, as tumor cells alter antigen expression to evade immune recognition (20). This antigen variability further diminishes the anti-tumor efficacy of CAR T-cells, as not all tumor cells can be targeted and eliminated. Therefore, it is evident that to achieve clinical success in the treatment of solid cancers, CAR T-cells will need to be engineered to be capable of efficiently bypassing all of these obstacles within the solid TME.

One strategy to overcome the barriers of the solid TME is to ‘armor’ CAR T-cells, typically by co-expressing a biological payload alongside the CAR that counteracts the immunosuppressive features of the TME (10, 11). A wide range of armoring approaches have been developed, many with pleiotropic effects that could fall into multiple functional categories. For the purposes of this review, we define armoring as the incorporation of a transgenic payload into CAR T-cells to enhance their anti-tumor activity. We broadly categorize armoring strategies into those that: 1) exploit cytokine signaling, 2) neutralize immune-inhibitory signals in the TME, 3) modulate metabolic pathways, 4) address antigen heterogeneity or antigen escape, and 5) improve CAR T-cell homing to tumors. As a comprehensive review of all armoring approaches is beyond the scope of this article, we particularly focus on recent or novel advances that have shown promising preclinical outcomes or have progressed to clinical evaluation (**Table 1**, **Supplementary Table S1**).

**Table 1:** Examples of armored CAR T-cells in clinical trials.

Armoring strategy	Phase of trial, target antigen, tumor types	Clinical trial identifier and references
IL-15	Phase I, GPC3, hepatocellular carcinoma	NCT02905188 (37)
	Phase I, GPC3, pediatric solid tumors	NCT02932956 (37)
	Phase I, GPC3, pediatric solid tumors	NCT04377932 (37)
	Phase I, GPC3, solid tumors	NCT05103631 (37)
	Phase I, GD2, neuroblastoma, osteosarcoma	NCT03721068
IL-15 + IL-21	Phase I, GPC3, pediatric solid tumors	NCT04715191
IL-12	Phase I, MUC16^ecto^, high-grade serous ovarian cancer	NCT02498912 (48)
	Phase I, EGFR, colorectal cancer	NCT03542799
IL-18	Phase I, GD2, neuroblastoma, breast cancer, Ewing sarcoma, osteosarcoma	EU CT 2022–501725–21–00
IL-7 receptor	Phase I, GD2, pediatric brain tumors	NCT04099797 (69)
	Phase I, GD2, neuroblastoma and other solid tumors	NCT03635632
dnTGF-βRII	Phase I, PSMA, prostate cancer	NCT03089203 (87)
	Phase I, PSMA, prostate cancer	NCT04227275 (88, 89)
	Phase I, claudin18.2, gastrointestinal adenocarcinomas	NCT05981235
anti-PD-1/PD-L1	Phase I, mesothelin, solid tumors	NCT04503980 (104)
	Phase I, mesothelin, solid tumors	NCT05089266 (104)
	Phase I/II, mesothelin, solid tumors	NCT03615313 (104)
Bispecific T-cell engagers	Phase I, EGFRvIII, glioblastoma	NCT05660369 (154)
CXCR1/CXCR2	Phase I, CD70, glioblastoma	NCT05353530
CXCR5	Phase I, EGFR, non-small cell lung cancer	NCT05060796
CCL19 + IL-7	Phase I, GPC3, solid tumors	NCT04405778 (198)
CCL19 + IL-7	Phase I, GPC3 or mesothelin, hepatocellular carcinoma, pancreatic cancer, ovarian cancer	NCT03198546 (199)

## Exploiting cytokine signaling to enhance CAR T-cell potency and rewire the TME

**Figure 3:**
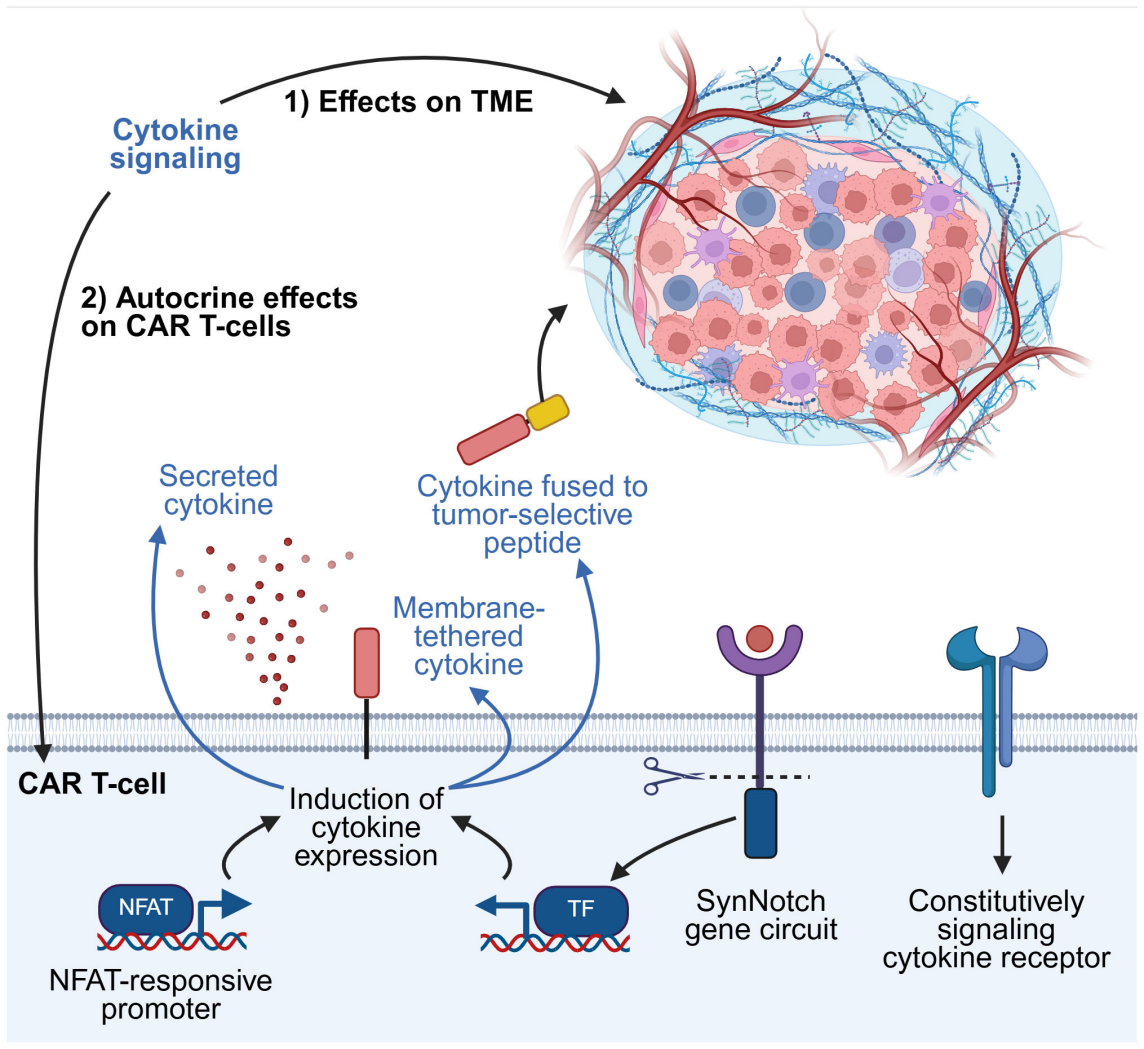
Strategies for exploiting cytokine signaling. Harnessing cytokine signaling pathways can induce beneficial effects by modulating the TME, the CAR T-cells themselves, or both. Cytokines can be secreted as soluble factors from CAR T-cells into the TME. Selected examples of other methods of regulating cytokine expression are illustrated here, including cytokines tethered to the CAR T-cell membrane or fused to tumor-selective peptides, modified cytokine receptors that signal constitutively but do not respond to native extracellular cytokine, and synthetic gene circuits. TF, transcription factor. This figure was created using BioRender.com.

T-cells Redirected for Universal Cytokine Killing (TRUCKs) represent some of the earliest examples of armored CAR T-cells (21–23). This term typically refers to CAR T-cells engineered to secrete pro-inflammatory cytokines that enhance anti-tumor activity by promoting CAR T-cell survival and effector functions, or by modulating the immunosuppressive TME (11, 24). More recently, novel armoring strategies have expanded beyond this classical definition, exploring alternative methods of inducing cytokine signaling within CAR T-cells, or incorporating cytokines that provide functional benefits unrelated to traditional inflammatory pathways (**Figure 3**).

### Common γ-chain cytokines

The common γ-chain (γ_c_) family of cytokines comprise IL-2, -4, -7, -9, -15, and -21, which all signal through receptors containing a shared γ_c_ subunit, and play crucial roles in lymphocyte proliferation, differentiation, and homeostasis (25). As such, this family of cytokines has received significant attention as potential armoring payloads for CAR T-cells.

Among these, IL-15 has emerged as a particularly promising candidate. IL-15-armored CAR T-cells have been demonstrated to exhibit enhanced proliferation, improved anti-tumor efficacy, sustained killing upon repeated tumor challenges *in vitro* and *in vivo*, prolonged survival in tumor-bearing mice, and promotion of central memory or stem cell memory-like phenotypes (26–30). In one study using a syngeneic melanoma mouse model, IL-15-armored CAR T-cells not only improved intrinsic T-cell function but also beneficially remodeled the TME, enhancing natural killer (NK) cell activation and reducing immunosuppressive ‘M2’ macrophage abundance (29). Another study found that CAR T-cells modified to fuse IL-15 to the scFv portion of the CAR could deplete immunosuppressive MDSCs in murine glioblastoma models, based on the observation that MDSCs in these models as well as human glioblastoma samples express the α subunit of the IL-15 receptor. This MDSC depletion was associated with reduced tumor growth and improved survival of the mice (31).

Despite these encouraging findings, concerns have been raised about the clinical translation of IL-15 armoring due to toxicities observed in trials of recombinant human IL-15, as well as some studies of IL-15-armored CAR T-cells in mice, including cytokine release syndrome (CRS), thrombocytopenia, and liver toxicity (32–36). To address safety concerns, several groups have developed regulatory mechanisms to try to prevent these toxicities. For example, Chen et al. incorporated an inducible caspase-9 (iC9) suicide gene into their IL-15-armored CAR construct. They demonstrated that administration of a chemical inducer of dimerization (CID) drug, which causes the iC9 protein to dimerize and activate apoptosis, enabled rapid elimination of the CAR T-cells (26).

IL-15-armored CAR T-cells, including those with the iC9 suicide gene, have now been evaluated in phase I trials. In one study, 4 out of 12 patients with glypican-3 (GPC3)-positive solid tumors achieved a partial response following treatment with IL-15-armored GPC3-targeting CAR T-cells, whereas none of the patients treated with unarmored CAR T-cells experienced an objective response (37). Notably, IL-15-CAR T-cell treatment was associated with a higher incidence of CRS that necessitated intervention with IL-1 or IL-6 blockade. In three patients with refractory CRS, activation of the iC9 switch successfully reduced circulating CAR T-cells and alleviated symptoms. Interestingly, this trial also reported an increase in effector memory and a decrease in central memory T-cell subsets among IL-15-armored CAR T-cells (37), an observation that contrasts with prior preclinical findings.

Another approach to circumvent the toxicities associated with constitutive IL-15 expression is to place IL-15 expression downstream of antigen-dependent T-cell activation. Ma et al. engineered CAR T-cells with IL-15 expression controlled by the interferon (IFN)-γ gene promoter, which activated upon TCR signaling. This antigen-dependent expression system led to selective IL-15 expression following tumor recognition and improved tumor control in a xenograft mouse model of gastric cancer (28).

The combination of two γ_c_ cytokines, IL-15 and IL-21, has also been tested in the preclinical setting, based on their known synergistic effects on cytotoxic lymphocytes (38, 39). CAR T-cells armored with both cytokines demonstrated superior anti-tumor activity in *in vivo* models compared to unarmored or single cytokine-armored CAR T-cells, and showed enhanced proliferation, increased frequencies of stem cell memory and central memory populations (40), and reduced exhaustion following repeated antigen stimulation (41).

### IL-12 family of cytokines

The IL-12 family comprises heterodimeric cytokines with pleiotropic roles in immune regulation. Among these, IL-12 and IL-23, members of the family which are considered to have predominantly pro-inflammatory effects (42), have garnered particular interest as potential armoring payloads for CAR T-cells.

IL-12 consists of two subunits, a p35 and a p40, and is primarily produced by antigen-presenting cells (APCs) (42). It promotes IFN-γ secretion from T-cells and NK cells, which in turn can further stimulate IL-12 release by macrophages and other APCs (42, 43). As an armoring payload, IL-12 has been demonstrated to enhance CAR T-cell effector functions and reprogram endogenous immune cells in the TME toward a pro-inflammatory, anti-tumor state (23, 42, 44). Although early efforts to armor CAR T-cells with IL-12 showed improved anti-tumor activity, constitutive or systemic expression of IL-12 has been associated with significant toxicities (45–48). In a phase I trial of an IL-12-secreting CAR T-cell therapy, two-thirds of patients who also received lymphodepleting chemotherapy developed dose-limiting hemophagocytic lymphohistiocytosis or macrophage activation-like syndrome (48).

More recent efforts to develop IL-12-armored CAR T-cells have therefore also endeavored to improve their safety profile by restricting IL-12 expression to the TME. One solution has been to bind IL-12 to the membrane of CAR T-cells (49, 50), which Hu et al. combined with fusing the IL-12 to a peptide targeting cell surface vimentin, a protein overexpressed by various solid tumors (49). These studies demonstrated that, compared to unarmored CAR T-cells, membrane-bound IL-12-CAR T-cells released more IFN-γ, and demonstrated superior anti-tumor efficacy in murine models of solid tumors, including large established tumors, without inducing systemic toxicity. These effects were accompanied by changes in immune cell populations in the TME, including enhanced dendritic cell (DC) maturation (49, 50). Lee et al. confirmed these findings in a model of ovarian cancer peritoneal metastasis, where systemic IL-12 administration caused toxicity, but membrane-bound IL-12-armored CAR T-cells did not (50).

Hombach et al. inserted IL-12 into the extracellular domain of the CAR. This configuration modulated CAR T-cells toward an NK cell-like phenotype, enabling them to kill both antigen-positive and antigen-negative tumor cells. This was demonstrated both *in vitro* and in a mouse model of CEA-negative ovarian cancer, where IL-12-armored CAR T-cells were effective, while conventional unarmored CEA-targeted CAR T-cells were not (51).

Other methods of achieving tumor-restricted IL-12 expression include: fusing IL-12 to a collagen-binding domain to localize it to exposed collagen in tumor vasculature and stroma (52); fusing it to a tumor-specific scFv (53); and placing IL-12 expression under control of a nuclear factor of activated T-cells (NFAT)-responsive promoter to link cytokine secretion to CAR activation (54, 55).

IL-23 is a heterodimeric cytokine that shares the same p40 subunit as IL-12, but pairs this with a p19 subunit. Ma et al. discovered that due to upregulation of p19 expression in T-cells upon activation, transducing T-cells with the p40 subunit was sufficient to induce IL-23 secretion from T-cells. They demonstrated that IL-23-secreting CAR T-cells exhibited improved tumor killing and reduced functional exhaustion compared to unarmored CAR T-cells in syngeneic mouse models of solid tumors. Notably, IL-23 acted via autocrine signaling to enhance CAR T-cell function. When compared to other armoring strategies, IL-23-armored CAR T-cells did not induce weight loss in immunodeficient mice, suggesting a superior safety profile relative to IL-15 and IL-18-armoring (56).

In contrast, another study found that PSMA-targeted CAR T-cells engineered to co-express an IL-23-targeting monoclonal antibody (mAb) led to eradication of prostate cancer in a murine model, suggesting that IL-23 blockade, not supplementation, may be beneficial in some contexts (57). These findings illustrate that the benefits of armoring can be context-dependent, influenced by tumor type, the immune microenvironment, and the preclinical model being used. Moreover, IL-23 is a multifaceted cytokine with reported pro-tumoral and anti-tumor effects, which may reflect differences in cytokine concentration (56, 58). These observations underscore the importance of considering not only the CAR T-cell dose, but also the ‘dose’ of the armoring payload when evaluating therapeutic efficacy and safety, which are critical factors for clinical translation.

### Other cytokines

#### Engineered IL-2 and IL-33

Although IL-2 is the prototypical T-cell growth factor and the first member of the γ_c_ family of cytokines to be discovered (25), its use as a payload has not gained significant traction due mainly to concerns about systemic toxicity. One group found that armoring CAR T-cells with superkine IL-2 (Super2), a variant of IL-2 that binds to the IL-2 receptor with higher affinity than the wild-type cytokine, and IL-33 significantly enhanced anti-tumor activity *in vivo* compared to armoring with either cytokine alone, without evidence of toxicity in the immunocompetent mouse models used (59). The rationale for armoring with IL-33 arose from studies that found that IL-33 activates intratumoral group 2 innate lymphoid cells (ILC2s) which can contribute to tissue-specific tumor immunity (59–61). The synergistic improvement in anti-tumor activity induced by Super2 and IL-33 armoring was independent of IFN-γ or perforin-mediated cytotoxicity. Instead, the combination appeared to function primarily by harnessing an endogenous immune response. This included increased tumor infiltration of endogenous T-cells and a shift toward pro-inflammatory ‘M1’ macrophage polarization, highlighting a potential strategy to remodel the TME and enhance CAR T-cell efficacy through immune orchestration rather than direct cytotoxic mechanisms (59).

#### IL-18

Like IL-12, IL-18 is a pro-inflammatory IFN-γ inducer, and has been found to have beneficial effects as an armoring payload. These include enhancing the proliferation and survival of CAR T-cells and modulating the TME to recruit and polarize endogenous immune cells to support the anti-tumor response (62–64). Jaspers et al. compared murine IL-18-armored, IL-12-armored and unarmored CAR T-cells in a syngeneic mouse metastatic model of small cell lung cancer. They found that only IL-18-armored CAR T-cell treatment induced tumor shrinkage, which was associated with improved survival. Moreover, phenotypic analysis of CAR T-cells post-injection into tumor-bearing mice revealed increased memory marker and reduced exhaustion marker expression by IL-18-secreting CAR T-cells compared to unarmored CAR T-cells (64).

As with other pro-inflammatory cytokine payloads, concerns about potential toxicities that may result from constitutive IL-18 signaling have led to efforts to regulate its expression (63, 65). Hull et al. demonstrated that constitutive expression of murine IL-18 by CAR T-cells induced lethal toxicity in mice, with postmortem blood analysis indicating CRS (65). To regulate IL-18 activity, they modified the cleavage site within pro-IL-18, the biologically inactive precursor, to one recognized by granzyme B, a cytotoxic protease released by T-cells. This design restricted IL-18 activation to the context of CAR T-cell activation and granzyme B release. They confirmed that this strategy enhanced CAR T-cell anti-tumor activity both *in vitro* and *in vivo*, without inducing significant toxicity in the same mouse model (65). Other strategies to link IL-18 expression to CAR T-cell activation have also been explored, including NFAT-responsive promoter systems, one of which is being evaluated in a first-in-human phase I trial against GD2-positive tumors (63, 66).

Lange et al. took a different approach by engineering a chimeric cytokine receptor consisting of the extracellular domain of the granulocyte-macrophage colony-stimulating factor (GM-CSF) receptor fused to the transmembrane and intracellular domains of the IL-18 receptor. Since GM-CSF is secreted by CAR T-cells upon antigen engagement, its binding to this chimeric receptor triggered activation-dependent IL-18 signaling within the CAR T-cells. This system was shown to induce tumor regression in two tumor xenograft models in which unarmored CAR T-cells were ineffective (67).

### Modifying cytokine receptors

Apart from engineering CAR T-cells to secrete or express cytokines, another strategy to exploit cytokine signaling is to modify the cytokine receptors. This approach has been applied particularly to cytokines of the γ_c_ family and circumvents the off-target effects that can result from transgenic cytokine expression, which may activate signaling on bystander cells in addition to the CAR T-cells themselves. Shum et al. developed a constitutively signaling IL-7 receptor, termed C7R, which activated the IL-7 signaling axis in CAR T-cells but was unresponsive to extracellular IL-7. They demonstrated that C7R-expressing CAR T-cells were more resilient to repeated tumor challenges, and capable of controlling or clearing tumors in several *in vivo* models (68). A phase I clinical trial of C7R-CAR T-cells demonstrated tolerability, with mostly grade 1 inflammation-associated toxicities, apart from one case of grade 4 CRS, and reported partial responses in 2 out of 7 patients with diffuse midline glioma (69). Another approach to harnessing IL-7 signaling has been to incorporate a portion of the IL-7 receptor-α intracellular domain into the CAR structure itself, which was demonstrated to enhance T-cell proliferation and improve tumor control (70, 71).

An additional strategy to avoid off-target effects is the design of mutated, or orthogonal, cytokine and receptor pairs that interact exclusively with each other, and not with their native counterparts. This concept was first demonstrated using an orthogonal IL-2 and IL-2 receptor pair (72). Following on from this, Kalbasi et al. engineered T-cells to express a synthetic cytokine receptor ‘o9R’, which combines the extracellular domain of the orthogonal IL-2 receptor with the intracellular domain of the IL-9 receptor. Upon stimulation with orthogonal IL-2, o9R signaling induced features consistent with a stem cell memory phenotype in the T-cells, an attribute potentially beneficial for CAR T-cell therapy. CAR T-cells expressing o9R and receiving orthogonal IL-2 also exhibited greater anti-tumor activity in syngeneic mouse models compared to CAR T-cells expressing the orthogonal IL-2 receptor (73).

More recently, Bell et al. replaced the extracellular domains of several heterodimeric cytokine receptors, including those of γ_c_ cytokines, and IL-10 and IL-12 receptors, with heterodimerizing leucine zipper motifs. This modification led to constitutive signaling through these receptors. CAR T-cells expressing these leucine zipper-modified cytokine receptors showed improved cytotoxicity in response to multiple tumor challenges *in vitro*, and CAR T-cells with a modified IL-2 receptor demonstrated superior anti-tumor activity *in vivo* in mouse xenograft models of lung cancer and sarcoma (74).

### Synthetic cytokine circuits

Designing synthetic circuits to restrict cytokine production specifically within the tumor is another strategy aimed at preventing systemic cytokine release and thereby reducing toxicity. Synthetic Notch (synNotch) receptors have been a pioneering example of synthetic gene circuits in the CAR field. These receptors consist of a synthetic antigen-recognizing extracellular domain, a transmembrane domain derived from the Notch signaling receptor, and a synthetic intracellular transcription factor. When the extracellular domain binds its cognate antigen, the synNotch receptor undergoes transmembrane cleavage, releasing the transcription factor to activate expression of chosen target genes placed downstream of a synthetic promoter (75). While the downstream payload can, in principle, be any gene of interest, synNotch-controlled cytokine release provides a compelling example of the utility of this system. Allen et al. demonstrated that CAR T-cells co-expressing a synNotch receptor designed to drive IL-2 production were able to eradicate solid tumors without inducing significant toxicity (76).

## Combating immune-inhibitory signals in the TME

**Figure 4:**
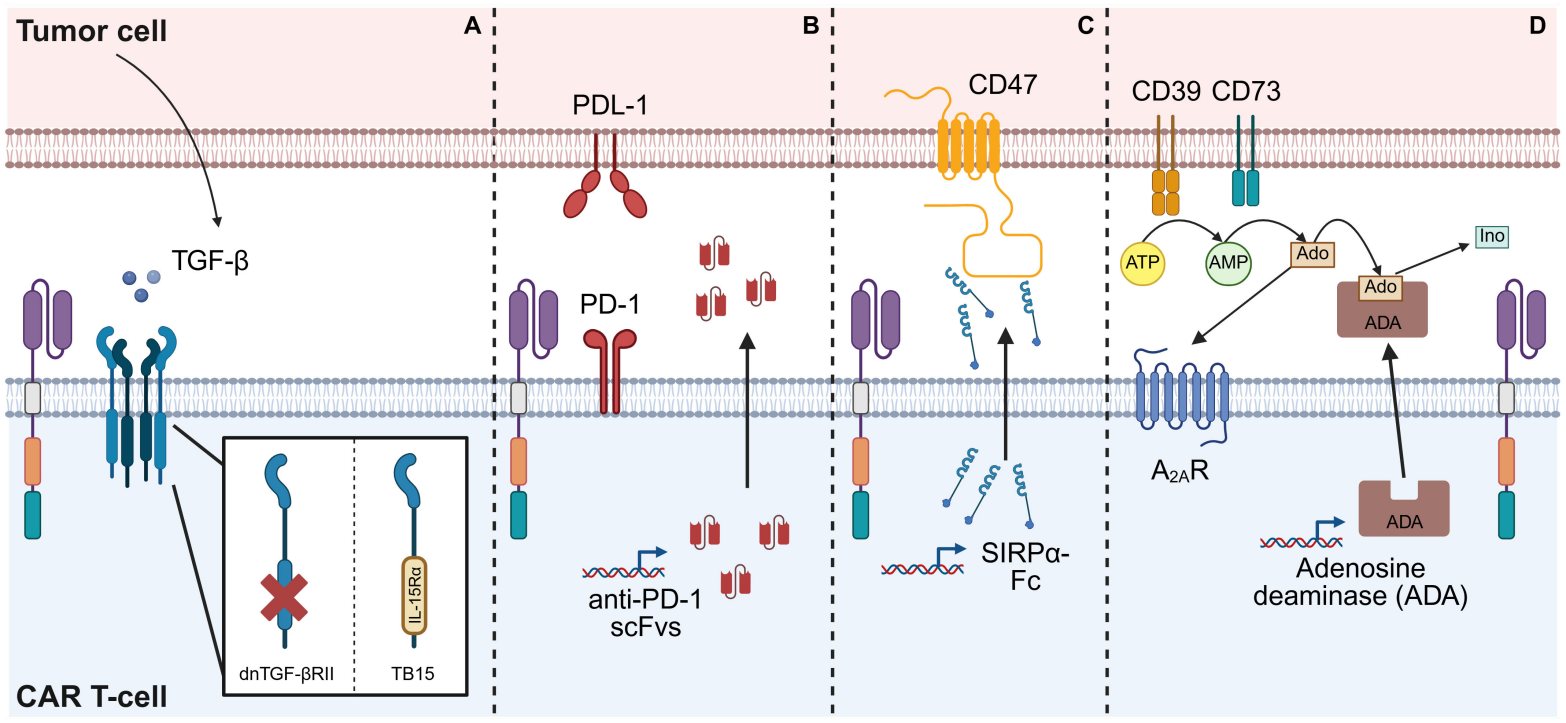
Strategies for combating immune-inhibitory signals in the TME. **(A)** TGF-β released within the TME can suppress CAR T-cell functions. Armoring a CAR T-cell with a ‘dominant-negative’ TGF-β receptor II (dnTGF-βRII), which features a truncated intracellular domain, prevents downstream signaling, thereby shielding the CAR T-cells from TGF-β-induced suppression. Alternatively, this intracellular domain can be replaced with that of a different receptor, such as IL-15 receptor-α, resulting in a chimeric receptor (TB15) which induces IL-15 signaling. **(B)** Binding of PD-L1 expressed on the surface of tumor cells to the PD-1 receptor on CAR T-cells suppresses CAR T-cell proliferation and effector functions. One method of preventing this is by engineering the CAR T-cells to secrete scFvs which neutralize PD-1 both on the CAR-T cells themselves and on adjacent cells, thereby blocking this inhibitory pathway. **(C)** CD47 expressed on the surface of tumor cells provide a ‘don’t eat me’ signal to APCs, protecting the tumor cells from phagocytosis. A strategy to overcome this is to engineer CAR T-cells to secrete molecules, such as a SIRPα-Fc fusion protein, that block CD47. **(D)** Adenosine (Ado) produced by breakdown of excess ATP within the TME can limit CAR T-cell functions by signaling through A_2A_R. Armoring CAR T-cells to release the enzyme adenosine deaminase (ADA) results in the catabolism of adenosine to inosine (Ino), thereby reducing the immunosuppressive effect of adenosine. This figure was created using BioRender.com.

CAR T-cells face numerous immunosuppressive signals within the TME, which can lead to functional exhaustion and diminished effector activity, ultimately resulting in reduced or lost therapeutic efficacy. To address this, armoring CAR T-cells with payloads that block or counteract these immune-inhibitory signals represents a promising strategy to enhance their anti-tumor potential (**Figure 4**).

### TGF-β

Although TGF-β is a cytokine with pleiotropic effects, it has drawn particular interest in the CAR T-cell field for its tumor-promoting and immune-inhibitory roles in the TME of many solid tumors, especially its suppression of T-cell effector functions (77, 78). Kloss et al. aimed to counteract this by armoring CAR T-cells with a ‘dominant-negative’ TGF-β receptor II (dnTGF-βRII), a truncated receptor lacking the intracellular signaling domain, which acts as a decoy to sequester TGF-β and prevent signaling in both CAR T-cells and neighboring immune cells. These dnTGF-βRII-armored CAR T-cells were resistant to TGF-β-induced suppression and exhibited enhanced proliferation and improved anti-tumor efficacy in a xenograft mouse model of prostate cancer (79). Enhanced efficacy with dnTGF-βRII armoring has since been reported in preclinical models of prostate cancer (80), ovarian cancer (81, 82), pancreatic cancer (83, 84), esophagogastric cancers (84), and glioblastoma (85). Notably, Tran et al. showed that their ROR1-targeting CAR T-cells induced TGF-β expression in tumor cells that did not initially overexpress this cytokine, but that this was mitigated by dnTGF-βRII armoring, which restored anti-tumor activity (83). A further advanced version of this strategy replaces the intracellular domain of TGF-βRII with that of another receptor, such as IL-15 receptor-α, thereby simultaneously blocking TGF-β and inducing IL-15 signaling. This dual-function receptor, named TB15, was shown to enhance CAR T-cell function beyond dnTGF-βRII or IL-15 armoring alone (86).

A dnTGF-βRII-armored PSMA-targeting CAR T-cell has since been evaluated in a phase I trial against metastatic castration-resistant prostate cancer. Grade 2 or higher CRS occurred in 5 out of 13 patients and was mostly manageable with immunosuppressive therapy, although one patient developed grade 4 CRS and concurrent sepsis and subsequently died. Four patients experienced ≥30% reductions in prostate-specific antigen levels, although the best radiographic response was stable disease (87). In a second phase I trial, two of nine patients experienced fatal immune-mediated toxicities, leading to early trial termination (88, 89). Retrospective analyses could not conclusively explain these severe toxicities, although the investigators speculated that modifying the co-stimulatory domain by replacing 4-1BB might help mitigate cytokine release without impairing efficacy (89). These findings underscore the gap between promising preclinical outcomes and complex clinical realities, possibly due to the widespread use of immunodeficient mouse models, which fail to fully capture human immune responses and associated toxicities.

An alternative strategy for blocking TGF-β signaling involves engineering CAR T-cells with an extracellular TGF-β-binding scFv. One group found that this approach paradoxically converted TGF-β into a stimulatory signal that enhanced CAR T-cell proliferation and activated immunostimulatory pathways (90). This group subsequently developed a bispecific CAR, targeting both IL-13Rα2 and TGF-β, which improved survival in glioblastoma mouse models (91). Other approaches for inhibiting TGF-β signaling include SMAD7-expressing CAR T-cells (92, 93), and knockout of the endogenous TGF-βRII in CAR T-cells using CRISPR/Cas9 technology (94), both of which improved anti-tumor efficacy *in vivo*.

### PD-1/PDL-1

A major factor limiting CAR T-cell efficacy within the TME is exhaustion, which can involve multiple signaling pathways, including activation of immune checkpoints that suppress CAR T-cell proliferation, cytotoxicity and other effector functions (95, 96).

PD-1, a cell surface receptor on T-cells, is one such inhibitory immune checkpoint; its sustained expression or upregulation is widely regarded as a hallmark of exhaustion (97). An armoring strategy that has gained significant interest involves engineering CAR T-cells that secrete anti-PD-1 scFvs, thereby blocking the PD-1/PD-L1 axis within the TME (95). This approach has been demonstrated to enhance CD107a expression (a marker of degranulation), as well as secretion of effector molecules, such as IFN-γ and granzyme B, by both CAR T-cells and endogenous tumor-specific T-cells, suggesting a broader restoration of anti-tumor immunity (98–100). Anti-PD-1 scFv-secreting CAR T-cells have demonstrated improved anti-tumor activity in preclinical *in vivo* models of non-small cell lung cancer (NSCLC) (98), breast cancer (101), ovarian cancer (99), and hepatocellular carcinoma (HCC) (100). Similarly, CAR T-cells that secrete antibodies that block PD-L1, the ligand for PD-1, have demonstrated enhanced efficacy in models of pancreatic cancer (102) and renal cell carcinoma (RCC) (103). In a humanized RCC mouse model, Wang et al. reported that tumors treated with anti-PD-L1 antibody-secreting CAR T-cells exhibited increased endogenous T-cell infiltration and a reduction in M2-like macrophages, indicating TME modulation by the secreted payload (103). Based on such promising preclinical results, several anti-PD-1 or anti-PD-L1-armored CAR T-cells have entered phase I clinical trials. One report described CAR T-cells with IFN-γ-induced secretion of anti-PD-1 nanobodies achieving partial responses in 6 out of 11 patients with mesothelin and PD-L1-positive mesotheliomas, as well as one complete response, and toxicities that were manageable with supportive care (104). Further reports from early phase clinical studies are awaited.

Combinations of PD-1 or PD-L1 blockade with additional mechanisms to enhance CAR T-cell function or modulate the TME have also been explored. For example, one group armored CAR T-cells to secrete a bispecific scFv targeting both PD-1 and TREM2 (105), an immunoglobulin receptor expressed on immunosuppressive TAMs and MDSCs (105, 106). These dual-targeting CAR T-cells outperformed those secreting scFvs against either PD-1 or TREM2 alone *in vivo*, and were associated with decreased M2 macrophages and MDSCs, and increased proportion of CD8^+^ T-cells (105). Another strategy combined PD-1 and TGF-β pathway inhibition via secretion of a bifunctional ‘trap’, a fusion of an anti-PD-1 scFv with the TGF-βRII ectodomain. In a xenograft model of prostate cancer, these dual-inhibition CAR T-cells suppressed tumor growth more effectively than unarmored or anti-PD-1-only armored CAR T-cells (107).

### CD47

Another immune checkpoint, often referred to as the ‘don’t eat me’ signal, involves CD47, a molecule frequently overexpressed on tumor cells. CD47 binds to the signal regulatory protein α (SIRPα) receptor on APCs, thereby inhibiting their phagocytic function (108). One strategy to counter this has been to engineer CAR T-cells to secrete a fusion protein, SIRPα-Fc, which blocks CD47. Chen et al. provided *in vitro* evidence that SIRPα-Fc enhanced macrophage phagocytic activity. *In vivo*, SIRPα-Fc-armored CAR T-cells exhibited improved anti-tumor efficacy and prolonged survival across several mouse models of solid tumors. This armoring also modulated immune cell phenotypes, promoting a T-cell central memory phenotype and increasing M1 macrophages and CD11c^+^ DCs in the TME (109).

Alternative strategies to target this axis have also been reported. Martins et al. armored CAR T-cells with a signal regulatory protein γ (SIRPγ)-related protein that binds to CD47 with high affinity, while Xie et al. engineered CAR T-cells to secrete CD47-specific nanobodies. In both cases, blocking the CD47 checkpoint enhanced CAR T-cell function and anti-tumor activity, including in models with tumor antigen heterogeneity (110, 111).

Interestingly, another group observed that concomitantly administering an anti-CD47 mAb with CAR T-cells depleted the CAR T-cells themselves secondary to macrophage-mediated phagocytosis, revealing a significant potential shortcoming of utilizing CD47 blockade as an armoring strategy (112). This issue was overcome by engineering CAR T-cells to co-express a variant of CD47 (CD47_E_) that retained SIRPα interaction but was resistant to blockade by anti-CD47 antibodies. Co-administering CD47_E_-CAR T-cells and a CD47-blocking mAb thereby protected the CAR T-cells from macrophage-mediated phagocytosis, whilst unleashing phagocytosis against tumor cells. In multiple *in vivo* models, CD47_E_-CAR T-cells administered alongside a CD47-blocking mAb exhibited increased persistence, enhanced macrophage infiltration into the TME, and improved anti-tumor activity (112).

### Adenosine

The solid TME is characterized by hypoxia, metabolic stress, and high cellular turnover, which leads to the accumulation of extracellular adenosine triphosphate (ATP). This ATP is converted to adenosine by CD39 and CD73, enzymes which are expressed on tumor cells and immunosuppressive cells within the TME. Activation of adenosine receptor 2A (A_2A_R) by adenosine represents another immune checkpoint that impairs anti-tumor immune responses, including limiting CAR T-cell effector functions (113–116).

Adenosine deaminase (ADA) is an enzyme that catabolizes adenosine into inosine, thereby preventing accumulation of adenosine. Qu et al. found that armoring CAR T-cells to secrete ADA increased the resistance of CAR T-cells to exhaustion, promoted intratumoral CAR T-cell expansion, and improved tumor control (117). Similarly, Hu et al. explored the benefits of armoring CAR T-cells with ADA, hypothesizing that this strategy would not only relieve adenosine-mediated immunosuppression, but also provide inosine as an alternative fuel to support CAR T-cell proliferation and function. To avoid supplying inosine to tumor cells, they engineered ADA-secreting CAR T-cells with the secreted ADA anchored to the CAR T-cell. This strategy resulted in enhanced CAR T-cell proliferation, higher inosine concentrations, greater T-cell infiltration into the TME, and improved anti-tumor activity (118).

Other strategies to combat adenosine-induced immunosuppression have also been explored, including knocking-down or deleting A_2A_R expression in CAR T-cells using short hairpin RNA (shRNA) (119) or CRISPR/Cas9 technology (120, 121). These approaches have been demonstrated to enhance CAR T-cell effector functions. As gene editing technologies like CRISPR/Cas9 continue to improve in safety and efficacy, the concept of armoring CAR T-cells may evolve beyond supplying one or two transgenic payloads to include more complex modifications to the CAR T-cell genome (122, 123).

### Tumor stroma: CAFs and the ECM

Apart from immune-inhibitory signals from tumor and immune cells, the activities of other stromal cells in the TME, such as CAFs, can also contribute to the suppression of CAR T-cell effector functions. CAFs secrete soluble factors, including immunosuppressive cytokines, and play a key role in producing the collagen-rich extracellular matrix (ECM) which create a physical barrier to CAR T-cell infiltration (18, 19, 124). One strategy to counteract CAFs is to target fibroblast activation protein (FAP), a pan-CAF marker, which some groups have achieved by engineering FAP-targeting CAR T-cells (125–127). One study found that treating pancreatic tumors, characterized by their dense desmoplastic stroma, in mice with FAP-targeting CAR T-cells reduced the integrity of the desmoplastic matrix, thereby making the tumors more susceptible to infiltration by CAR T-cells targeted against mesothelin, a TAA (126). Wehrli et al. also aimed to address CAF-associated immunosuppression by armoring mesothelin-targeting CAR T-cells to secrete a bispecific molecule targeting both CD3 and FAP, thereby inducing CAR-mediated killing of antigen-expressing tumor cells and engaging T-cells to kill CAFs. These armored CAR T-cells were shown to be effective against multiple models of pancreatic cancer (128). Notably, the FAP-targeted scFv used in this study did not cross-react with murine FAP, a challenge that the authors navigated by using several preclinical models, including patient-derived organoids with patient-matched CAFs, to characterize the armored CAR T-cells (128). This highlights one challenge that affects the study of armored CAR T-cells, which is that of potential cross-species barriers between the armoring payload and host cells in preclinical mouse models.

Another approach to combating the tumor stroma is to target the ECM, which is composed of macromolecules including collagens, proteoglycans, and glycoproteins (129). One strategy is to armor CAR T-cells with enzymes that degrade ECM components. Both heparanase-expressing CAR T-cells (130) and hyaluronidase-expressing CAR T-cells (131) have demonstrated increased tumor infiltration and enhanced anti-tumor activity, including when hyaluronidase and IL-7 armoring were combined (132). Moreover, Zheng et al. engineered CAR T-cells to express a synNotch receptor that drives the expression of three ECM-degrading enzymes, matrix metalloproteinase (MMP) 9, MMP12, and heparanase. They demonstrated that these synNotch CAR T-cells were more resistant to exhaustion and apoptosis after tumor challenge and exhibited enhanced tumor infiltration and anti-tumor activity *in vivo* (133).

## Optimizing CAR T-cell fitness by modulating metabolic pathways

**Figure 5:**
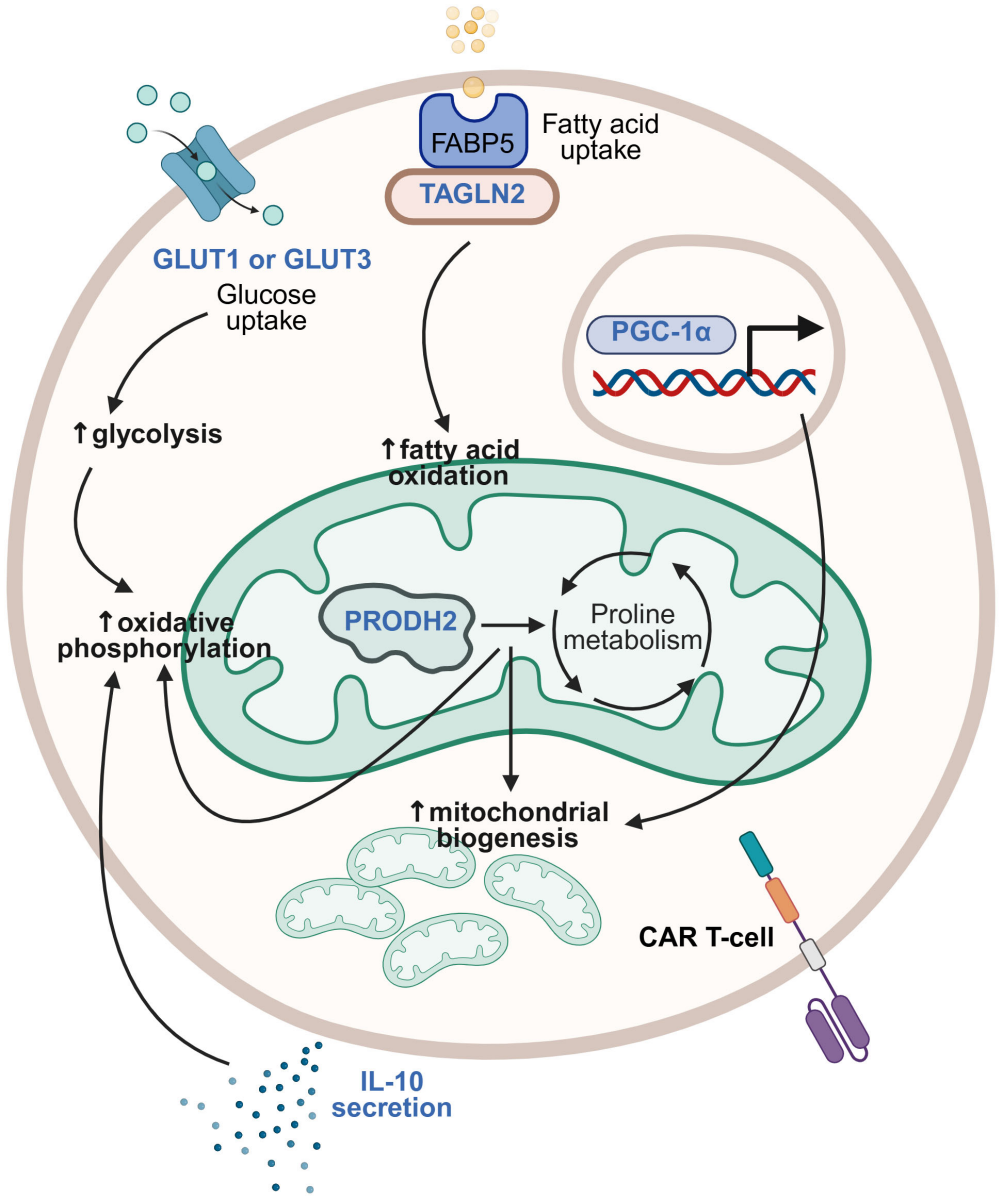
Strategies for modulating metabolic pathways. Different metabolic pathways may be modified in CAR T-cells to optimize their metabolic fitness. Metabolic armoring strategies that have been studied are illustrated here, with transgenic payloads (in blue) including glucose transporter (GLUT) 1 or GLUT3, transgelin 2 (TAGLN2), proline dehydrogenase 2 (PRODH2), IL-10, and PPARγ coactivator 1-α (PGC-1α) having been demonstrated to induce beneficial modulation of metabolic pathways including glycolysis, oxidative phosphorylation, and fatty acid oxidation, increased mitochondrial biogenesis, and associated improvement of CAR T-cell effector functions. This figure was created using BioRender.com.

Exploration of cellular metabolism and its pathways to identify novel targets for treating cancer has intensified over the past decade, although much remains to be elucidated in this complex field (134–136). Numerous groups have demonstrated that modulating CAR T-cell metabolism to overcome metabolic suppression in the TME can improve CAR T-cell survival and effector functions. Many of these strategies overlap with those designed to overcome immune suppression in the TME, as promoting immunostimulatory or blocking immune-inhibitory signals is often inextricably linked to the metabolic fitness of CAR T-cells. However, we review novel approaches for metabolic armoring separately here to facilitate discussion (**Figure 5**).

The solid tumor TME is an environment of intense competition for nutrients, with the tumor cells typically prevailing. Glucose uptake and glycolysis in tumor cells are enhanced, whilst other cells in the TME, including immune cells, experience metabolic starvation and dysfunction (137, 138). Modulating glucose metabolism in CAR T-cells is therefore an appealing strategy. There are 14 human glucose transporters (GLUTs) that mediate glucose transport into cells, with GLUT1 being the most widely expressed across cell types, and GLUT3 being a high-affinity isoform expressed primarily in neurons (139). Shi et al. reported the benefits of overexpressing GLUT1 in CAR T-cells, including increased proliferative capacity and persistence in low-glucose conditions, increased proportions of stem cell-like memory T-cells after repeated tumor challenge, upregulation of genes involved in glycolysis and oxidative phosphorylation, and improved anti-tumor activity *in vitro* and *in vivo* in models of both leukemia and solid tumors, including RCC and glioblastoma. They did not observe the same with GLUT3 overexpression in CAR T-cells against leukemia models, although testing of GLUT3 armoring in solid tumor models was not reported (140). In contrast, Hu et al. observed that GLUT3-armored CAR T-cells demonstrated increased uptake of the glucose analog 2NBDG, greater release of effector cytokines, and enhanced tumor cell killing *in vitro* compared to GLUT1-armored CAR T-cells. Moreover, GLUT3-CAR T-cells demonstrated superior anti-tumor activity compared to unarmored CAR T-cells in both xenogeneic and syngeneic mouse models of solid tumors, including pancreatic, esophageal, and lung cancer (141). The seemingly disparate results regarding the relative benefits of GLUT1 versus GLUT3 overexpression may not actually be conflicting, but stem from the use of different tumor models or variations in CAR T-cell types. Regardless, the heterogeneity of solid tumors and of their preclinical models means that specific armoring strategies may not be universally beneficial across all solid tumor types.

Lipid metabolism has also been targeted to enhance CAR T-cell effector functions. The fatty acid-binding protein 5 (FABP5)-fatty acid β oxidation (FAO) axis in T-cells has emerged as a potential therapeutic target due to FABP5’s role in facilitating the transport of exogenous fatty acids to the mitochondria for FAO and energy generation. Hwang et al. identified transgelin 2 (TAGLN2), a cytoskeletal actin-binding protein, as a key FABP5-binding partner required for FABP5 localization to the plasma membrane for fatty acid uptake. They discovered that TAGLN2 expression was downregulated in T-cells from cancer patients, due to tumor-induced endoplasmic reticulum (ER) stress responses. By overexpressing TAGLN2 in CAR T-cells to circumvent stress responses and maintain CAR T-cell fitness, they demonstrated that TAGLN2-armored CAR T-cells improved control of metastatic disease progression and survival of mouse models of ovarian cancer (142).

Amino acid metabolism has also been implicated in tumorigenesis and homeostasis, with proline being one amino acid that plays diverse roles within the TME (143, 144). Using a CRISPR activation screen to identify genes that could enhance the effector functions of CD8^+^ T-cells, Ye et al. identified proline dehydrogenase 2 (PRODH2) as a promising enzyme target. Overexpression of PRODH2 in CAR T-cells led to increased proliferative capacity, upregulation of CAR T-cell effector molecules, including IFN-γ and granzyme B, and downregulation of apoptosis. PRODH2-armored CAR T-cells also demonstrated higher mitochondrial mass, were shifted toward oxidative phosphorylation metabolism, and exhibited enhanced anti-tumor efficacy in a xenograft mouse model of breast cancer (145).

Cytokine signaling, the manifold benefits of which were discussed earlier, can also contribute to CAR T-cell armoring by improving their metabolic fitness. One recent study focused particularly on the metabolic benefits of armoring with IL-10 (146), traditionally considered to be an anti-inflammatory cytokine but also now recognized for its complex roles in cancer immunity (147, 148). Zhao et al. demonstrated that IL-10-armored CAR T-cells exhibited decreased mitochondrial dysfunction, enhanced oxidative phosphorylation, and a stem cell-like memory phenotype, as well as increased proliferative capacity and cytotoxicity. Furthermore, these CAR T-cells showed enhanced tumor control in various solid tumor mouse models, including rejection of tumor rechallenges in long-term survivors (146).

Finally, Lontos et al. addressed mitochondrial dysfunction in exhausted CAR T-cells by targeting PPARγ coactivator 1-α (PGC-1α), a master regulator of mitochondrial biogenesis. By overexpressing an inhibition-resistant variant of PGC-1α in CAR T-cells, they demonstrated that these CAR T-cells had increased effector cytokine expression, higher expression of stem cell-like memory markers, and enhanced anti-tumor activity (149).

Taken together, these studies highlight the potential benefits of modifying various metabolic pathways in CAR T-cells. Whether these benefits will translate into clinical success is an exciting area for future research.

## Overcoming antigen heterogeneity and antigen escape

**Figure 6:**
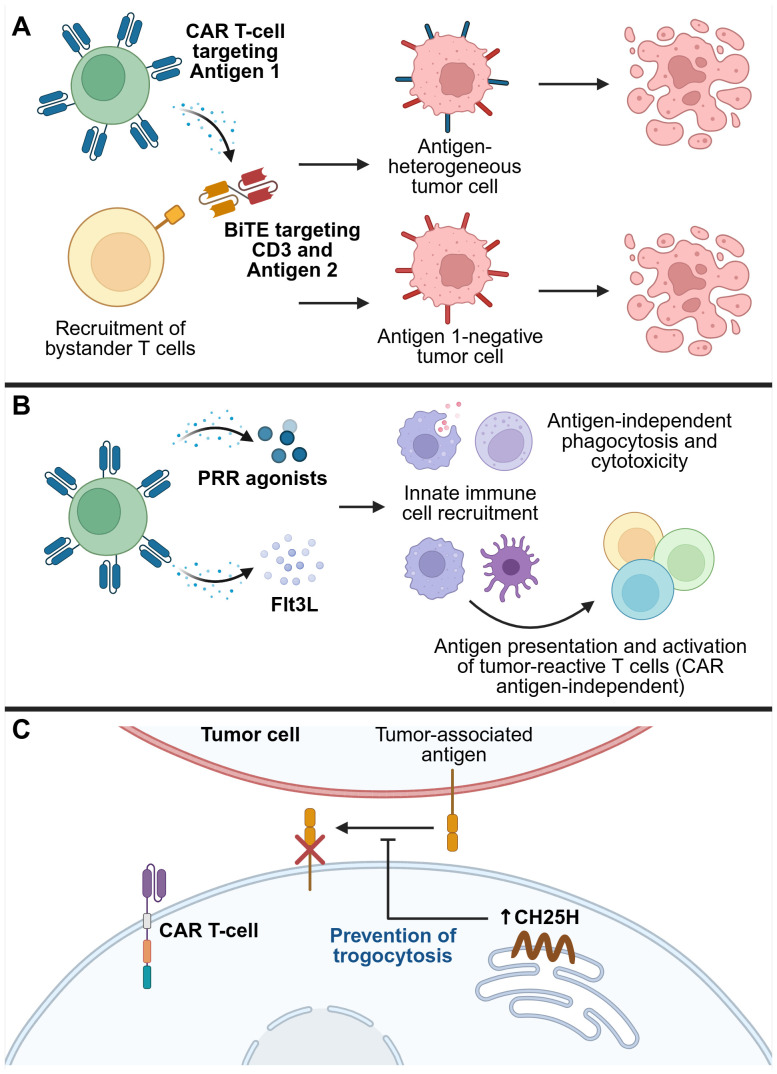
Strategies for overcoming tumor antigen heterogeneity and therapy escape mechanisms. **(A)** CAR T-cells secreting bispecific T-cell engagers (BiTEs) can both induce CAR-mediated tumor killing, and recruit bystander T-cells to kill tumor cells that do not express the CAR antigen. **(B)** Recruitment of endogenous innate immune cells, such as DCs, macrophages, and NK cells, activates their antigen-presenting, phagocytic, and cytotoxic capabilities to induce CAR antigen-independent tumor killing, as well as recruit the endogenous adaptive immune system, resulting in epitope spreading and broad anti-tumor immunity. **(C)** Restriction of trogocytosis can prevent the transfer of antigens from tumor cells to CAR T-cells, thereby preventing CAR T-cell fratricide and loss of efficacy. One mechanism that contributes to trogocytosis is upregulation of the transcription factor ATF3 by tumor exposure, which in turn suppresses the enzyme cholesterol 25-hydroxylase (CH25H). Overexpressing CH25H in CAR T-cells overcomes this issue, thereby reducing trogocytosis-mediated loss of antigen from tumor cells. This figure was created using BioRender.com.

Another variable that can impact CAR T-cell efficacy against solid tumors is the expression of antigen on tumor cells. A key challenge is the scarcity of bona fide TAAs that are entirely absent on healthy tissues and uniformly expressed across all tumor cells (150, 151). Additionally, antigen-negative tumor cells may become predominant through selective pressure, as CAR T-cells eliminate antigen-positive tumor cells. Tumors may develop mechanisms to downregulate the expression of the targeted antigen, resulting in a phenomenon known as ‘antigen escape’ in which CAR T-cells lose the ability to recognize and kill tumor cells (10, 150). One potential way to circumvent this issue is through recruitment of endogenous immune responses, particularly those mediated by innate immune cells that are not restricted to a single tumor antigen. This was previously discussed in the context of rewiring of the TME by cytokines such as IL-12, as well as the strategy of blocking CD47 to promote macrophage-mediated phagocytosis of tumor cells. Additional armoring strategies aimed at addressing antigen heterogeneity and escape are discussed below (**Figure 6**).

### Bispecific immune cell engagers

Bispecific T-cell engagers (BiTEs) are fusion proteins specific for two targets, one of which is an anti-CD3 scFv that engages T-cells, thereby enabling T-cell-mediated killing of cells expressing the second target (152). While BiTEs are being developed as standalone immunotherapeutics, engineering CAR T-cells to secrete BiTEs has also emerged as a strategy to overcome antigen heterogeneity. For example, Choi et al. engineered EGFRvIII-targeting CAR T-cells that secreted BiTEs against EGFR, and demonstrated *in vitro* that these armored CAR T-cells could recruit bystander T-cells to kill tumor cells. In orthotopic mouse models of glioblastoma with heterogeneous or negative EGFRvIII expression, these BiTE-armored CAR T-cells induced tumor regression (153). This strategy has since been tested in a first-in-human trial involving intraventricular delivery to three patients with recurrent glioblastoma, where it led to rapid tumor shrinkage. However, for two of the patients, the response was transient, underscoring the need for further optimization to enhance durability (154).

Similar strategies have been explored in other tumor types. These include: Muc16-targeting CAR T-cells armored with BiTEs against a peptide derived from the intracellular TAA Wilms tumor 1 (WT1), which demonstrated improved efficacy in ovarian cancer models with low Muc16 expression (155); and GPC3-targeting CAR T-cells that secrete BiTEs against B7-H3, which showed enhanced cytotoxicity against HCC cell lines with heterogeneous expression of GPC3 and B7-H3, although this was demonstrated only *in vitro* (156).

The repertoire of bispecific molecules that can be secreted by CAR T-cells continues to expand. Based on their findings of abundant CD16a-expressing innate immune cell infiltration in high-risk neuroblastoma TMEs, Pascual-Pasto et al. decided to engineer anti-glypican-2 (GPC2) CAR T-cells that secreted a bispecific innate immune cell engager (BiCE) targeting GD2 and CD16a, the latter serving to engage CD16a^+^ innate immune cells, including NK cells. They demonstrated that BiCE-armored CAR T-cells exhibited CAR-mediated cytotoxicity against GPC2-positive tumor cells and harnessed bystander NK cell activity against GD2-positive cells. This dual activity led to enhanced intratumoral NK cell retention and improved anti-tumor efficacy in patient-derived xenograft models of neuroblastoma in mice (157).

### Targeting PRRs to activate endogenous innate immunity

Damage-associated molecular patterns (DAMPs) and pathogen-associated molecular patterns (PAMPs) are molecular motifs recognized by pattern recognition receptors (PRRs), which can activate innate immune responses in an antigen-independent manner (158). As such, they offer a potential strategy for circumventing tumor antigen heterogeneity.

Toll-like receptors (TLRs) are an important class of PRRs, each receptor recognizing distinct PAMPs in a specific subcellular compartment (158). TLR5, for example, recognizes flagellin, a protein subunit of bacterial flagella. Flagellin can initiate pro-inflammatory signaling pathways via TLR5, which is expressed on several innate immune cell types including macrophages and DCs, and has therefore attracted attention for its potential use as a vaccine adjuvant (159). Several groups have also explored its use as an armoring payload in CAR T-cells. More recently, Niu et al. engineered CAR T-cells that secreted flagellin under the control of an NFAT-responsive promoter. While flagellin armoring did not enhance the cytotoxicity of CAR T-cells *in vitro*, it significantly prolonged tumor control and survival in immunocompetent mouse models of solid tumors, suggesting that its benefit lies in activating endogenous immune responses. Supporting this, the authors observed increased intratumoral abundance of CD86^+^ macrophages (indicative of M1 macrophage polarization) and CD103^+^ DCs (associated with DC activation). Additionally, in a model of antigen-heterogeneous melanoma, flagellin-armored CAR T-cells suppressed tumor growth more effectively than their unarmored counterparts (160).

Another bacterial protein explored as a CAR T-cell armoring payload is neutrophil-activating protein (NAP) from the bacterium *Helicobacter pylori*, which has been shown to exert various immunomodulatory effects. These include acting as a neutrophil chemoattractant and promoting IL-12 and IL-23 expression via TLR2 on neutrophils and monocytes, thereby supporting Th1 responses (161, 162). Jin et al. demonstrated that armoring CAR T-cells with NFAT promoter-inducible NAP improved anti-tumor activity in multiple syngeneic tumor models, including those with antigen-heterogeneity. NAP-armored CAR T-cells increased tumor infiltration of neutrophils, M1 macrophages and NK cells, and promoted ‘epitope spreading’ among endogenous CD8^+^ T-cells, that is, activation of CD8^+^ T-cells recognizing antigens other than the CAR-targeted TAA, suggesting robust bystander immune activation (163).

Beyond protein-based PRR agonists, nucleic acid-sensing pathways have also been leveraged. The cytosolic PRR RIG-I is activated by RNA and induces type I IFNs, which play broad roles in innate and adaptive immunity (164). Johnson et al. engineered CAR T-cells to secrete RN7SL1, a non-coding RNA that activates RIG-I, via extracellular vesicles that are preferentially taken up by immune rather than tumor cells. They found that delivery of RN7SL1 by the CAR T-cells promoted pro-inflammatory features and restricted immunosuppressive features among endogenous myeloid cells, and promoted the development of endogenous CD8^+^ T-cells with an effector memory-like phenotype. When combined with immune checkpoint blockade, RN7SL1-CAR T-cells eradicated antigen-heterogeneous melanomas in immunocompetent mice and prevented tumor outgrowth upon rechallenge with antigen-negative tumor cells, suggesting the generation of CAR antigen-independent memory (165).

Finally, PRR agonists as immune adjuvants have been combined with CAR T-cells secreting immune cell growth factors as another method of harnessing the endogenous immune system. Lai et al. armored CAR T-cells with Fms-like tyrosine kinase 3 ligand (Flt3L), a hematopoietic growth factor that is essential for steady-state DC development (166, 167), and demonstrated associated intratumoral expansion of conventional type 1 DCs, which play an important role in cross-presenting antigens to and activating cytotoxic CD8^+^ T-cell responses. When combined with poly(I:C), an immunostimulatory TLR3 agonist, Flt3L-secreting CAR T-cells significantly suppressed tumor growth in both antigen-homogeneous and heterogeneous tumor models. Treated mice also rejected antigen-negative tumor rechallenges, again indicating induction of epitope spreading and a durable anti-tumor immune response (168).

### Modulating trogocytosis

Trogocytosis is a phenomenon that has received increased attention in recent years within the field of immunology. It refers to the transfer of fragments of cell membrane, along with associated proteins and other molecules, between cells that are in direct contact with each other (169). Trogocytosis has been observed between tumor cells and CAR T-cells, leading to the transfer of TAAs from the tumor cells to the CAR T-cells. This can contribute to antigen escape and even CAR T-cell fratricide, where CAR T-cells inadvertently recognize and kill one another (170). In some cases, CAR molecules themselves can be transferred from CAR T-cells to tumor cells, further reducing anti-tumor efficacy (171). Lu et al. identified cholesterol metabolism as a key pathway involved in trogocytosis, potentially due to cholesterol’s role in modulating the biophysical properties of lipid membranes. They found that ATF3, a stress-responsive transcription factor, is upregulated in cytotoxic T lymphocytes (CTLs) exposed to tumor-derived signals. ATF3 suppresses expression of the enzyme cholesterol 25-hydroxylase (CH25H), which in turn promotes trogocytosis between CTLs and tumor cells, leading to T-cell dysfunction and tumor progression. By armoring CAR T-cells to co-express CH25H, they were able to reduce the extent of trogocytosis, preserve antigen expression on tumor cells, and enhance CAR T-cell infiltration and anti-tumor efficacy (172). Of note, other recent work has also examined how incorporation of different CAR transmembrane domains affect the probability of trogocytosis occurring, and how this can be used to modulate the effects of CAR T-cell therapy (173).

## Improving CAR T-cell homing to tumors

**Figure 7:**
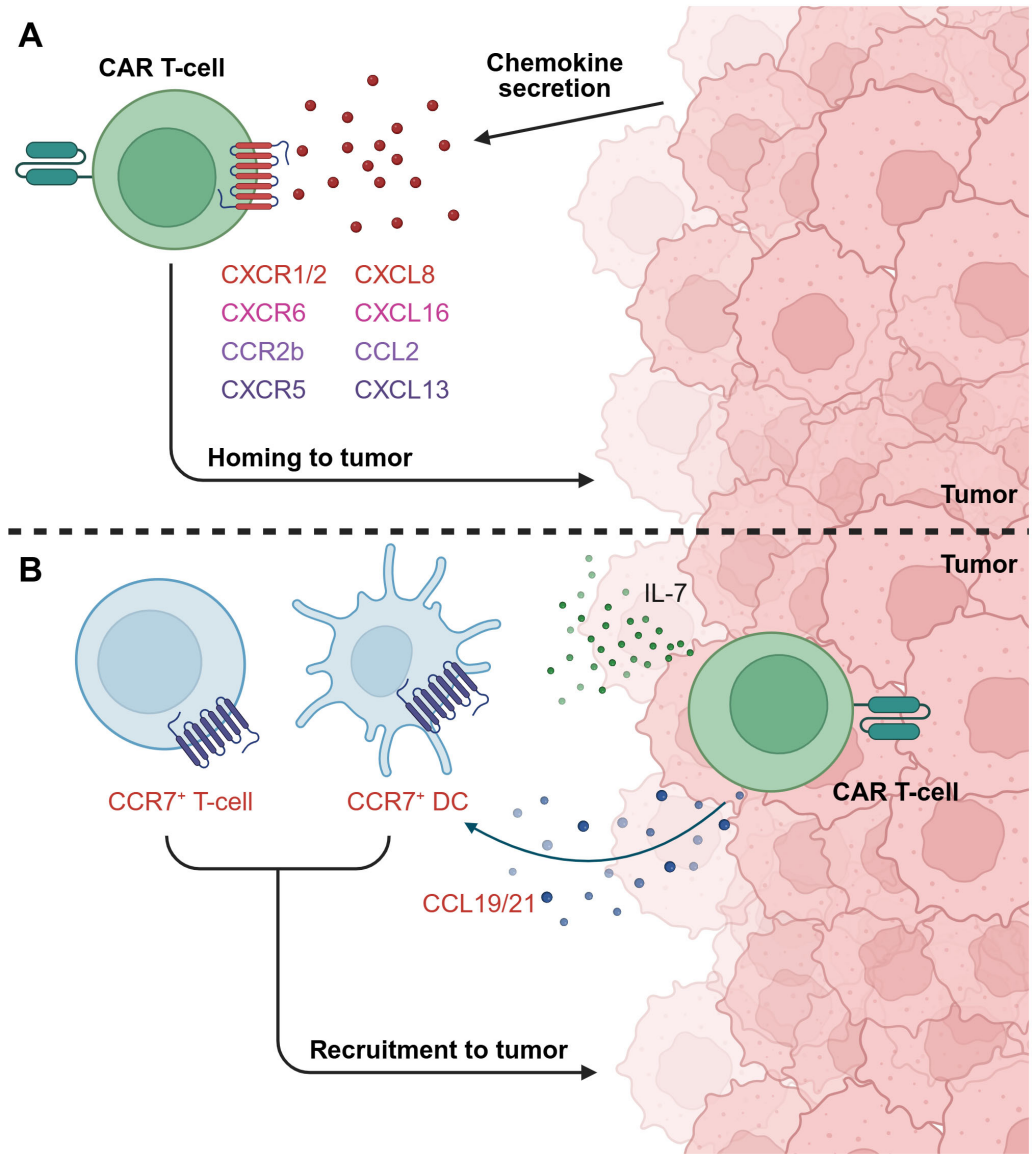
Exploiting chemokine/chemokine receptor signaling to improve CAR T-cell homing to tumors. **(A)** Armoring CAR T-cells with specific chemokine receptors, for example CXCR1/2, CXCR6, CCR2b, and CXCR5, can improve homing towards tumors that express the cognate chemokine ligand. **(B)** CAR T-cells can be engineered to secrete chemokines that recruit endogenous immune cells to support the anti-tumor response. For instance, illustrated here is a dual IL-7 and CCL19/21-armored CAR T-cell, which recruits T-cells and DCs expressing CCR7, the cognate receptor for CC19 and CCL21, to the TME. This figure was created using BioRender.com.

Enhancing the ability of CAR T-cells to efficiently home to tumors represents another important challenge to overcome. Systemically administered CAR T-cells can become sequestered at non-tumor-bearing tissues, and a lack of chemotactic cues can allow tumor cells to evade recognition and elimination by circulating CAR T-cells (174, 175). One strategy to address these limitations involves armoring CAR T-cells with chemokines or chemokine receptors selected to improve trafficking of CAR T-cells to tumor sites (**Figure 7**).

### Chemokine/chemokine receptor axes

CXCL8, also known as IL-8, is a chemokine associated with poor prognosis in numerous solid tumor types and with the recruitment of immunosuppressive immune cells to the TME. Several groups have sought to target this by armoring CAR T-cells with CXCR1 or CXCR2, both of which are CXCL8 receptors, with the aim of exploiting tumor CXCL8 expression to improve CAR T-cell migration to tumors (12, 176, 177). Notably, CXCL8 expression has been shown to be upregulated by irradiation of some tumors, suggesting the benefits of combining radiotherapy with CAR T-cell therapy in specific contexts (176, 177). CXCR1/CXCR2-armoring has been demonstrated to improve CAR T-cell infiltration, persistence, and tumor control *in vivo* across several studies (176–180). However, findings by Talbot et al. suggested that the benefits of CXCR2-armoring were context-dependent, as this did not enhance tumor control in a rapidly growing primary orthotopic xenograft model of osteosarcoma, but did confer benefits in a metastatic model (179). Interestingly, multiple groups have reported potentially improved safety profiles of CXCR-armored CAR T-cells compared to unarmored counterparts, possibly due to more targeted trafficking to tumors and thus fewer off-tumor toxicities (178, 179). On the other hand, one group found that while CXCR2 armoring enhanced trafficking into the TME, CAR T-cell persistence, and therefore anti-tumor efficacy, remained limited by the immunosuppressive TME. They demonstrated that co-armoring with the pro-inflammatory cytokines IL-15 or IL-18 could overcome this limitation, resulting in significantly improved *in vivo* anti-tumor activity (181).

Lesch et al. found CXCL16 to be highly expressed by both tumor and myeloid stromal cells in murine pancreatic cancer models and human specimens. Armoring CAR T-cells with CXCR6, the receptor for CXCL16, led to increased anti-tumor efficacy and prolonged the survival of treated mice (182). They also noted elevated expression of very late antigen-4 (VLA-4), an integrin that facilitates transendothelial migration of lymphocytes, in CXCR6-transduced T-cells, suggesting that harnessing the CXCL16-CXCR6 axis may offer multiple benefits for improving CAR T-cell trafficking. Talbot et al., who also tested CXCR6-armored CAR T-cells against osteosarcoma, found similar benefits in a metastatic xenograft model, and again reported an improved safety profile compared to unarmored CAR T-cells (179).

The CCL2-CCR2 axis has also drawn attention, as some solid tumors, including pleural mesothelioma and NSCLC, can secrete high levels of CCL2 (183, 184). CAR T-cells transduced with CCR2b, the dominant isoform of CCR2, showed improved tumor infiltration and tumor control in mouse models, including successfully crossing the blood-brain barrier to control brain metastases from NSCLC (183–185). On a similar theme, Li et al. armored CAR T-cells with CXCR5, the receptor for CXCL13, based on the expression of CXCL13 in a high proportion of NSCLC samples. These CAR T-cells exhibited enhanced migration toward CXCL13-positive targets and improved *in vivo* anti-tumor activity (186). Moreover, the combination of chemokine receptor (including CCR2b and CXCR5) and IL-7 armoring has been demonstrated to boost CAR T-cell expansion and anti-tumor activity in immunodeficient mouse tumor models (187, 188).

Tian et al. identified the CCL4/CCL5-CCR5 axis as one that is significantly correlated with CD8^+^ T-cell infiltration in solid tumors. They engineered CAR T-cells to express both CCR5 and IL-12, which enhanced T-cell trafficking and reversed TAM-induced immunosuppression (189). Conversely, the CCL1-CCR8 axis has been identified as having a role in the recruitment of immunosuppressive Tregs into the TME. Cadilha et al. armored CAR T-cells with both CCR8, to drive recruitment of CAR T-cells into the TME, and dnTGF-βRII, reasoning that this would counteract any Treg-mediated immunosuppression. They demonstrated that these dual-armored CAR T-cells significantly improved tumor control and survival *in vivo* (190).

Finally, chemokine armoring has also been found to be beneficial for recruiting other immune cells to the TME. Dual armoring with IL-7 and CCL19 has received particular focus, as these cytokines are essential for organizing the T-cell zone in lymphoid organs, and for recruiting T-cells and DCs expressing CCR7, the receptor for CCL19 (191, 192). Adachi et al. applied this mechanism to facilitate T-cell and DC recruitment to tumors (193), a strategy that has since also been studied by other groups. IL-7 and CCL19 dual armoring has been found to result in enhanced proliferation of the CAR T-cells, reduced expression of exhaustion markers, development of a central memory phenotype, remodeling of the TME, and improved *in vivo* control of tumors and survival in preclinical mouse models, including survival superior to armoring with either cytokine alone (193–196). Similar findings have been reported with dual armoring with IL-7 and CCL21, another CCR7 ligand, which also promoted T-cell and DC recruitment, increased central memory phenotype T-cells, and improved tumor control in an antigen-heterogeneous tumor model (197). Early phase clinical trials of dual IL-7 and CCL19-armored CAR T-cell have reported preliminary evidence of anti-tumor activity and no grade >2 toxicities (198, 199), although further studies will be needed to confirm clinical benefit.

Taken together, this wide assortment of studies have demonstrated the utility of exploiting chemokine signaling pathways to improve recruitment of CAR T-cells and other immune cells to solid tumors. However, the benefits of individual strategies are likely to be context-dependent and must be tailored according to the chemokine/chemokine receptor expression profile of the specific tumor being targeted. While chemokine receptor armoring alone has yielded enhanced anti-tumor effects in some instances, other studies suggest that additional payloads, such as pro-inflammatory cytokines, may be necessary to overcome the immunosuppressive TME and fully realize therapeutic potential.

### LIGHT

An armoring payload that potentially provides the benefits of both chemokine and pro-inflammatory signaling is LIGHT, also known as tumor necrosis factor superfamily member 14 (TNFSF14). Using RNA sequencing data from The Cancer Genome Atlas (TCGA) database, Zhang et al. identified a strong correlation between LIGHT expression and gene signatures of tertiary lymphoid structures (TLS) (200), structures in tumors that play key roles in recruiting immune cells and shaping immune responses (201). They demonstrated that CAR T-cells armored with LIGHT upregulated expression of chemokines CCL19, CCL21, and CXCL13 in both stromal cells and tumor cells, enhancing the ability of the stromal cells to recruit T-cells in migration assays. *In vivo*, LIGHT-armored CAR T-cells improved tumor infiltration, accelerated tumor regression, and prolonged survival in mice compared to their unarmored counterparts (200).

Separately, another group characterized the benefits of LIGHT-armoring from the perspective of overcoming tumor antigen heterogeneity (202). LIGHT binds to two receptors: lymphotoxin-β receptor (LTβR), which is broadly expressed on non-lymphoid cells, and herpesvirus entry mediator (HVEM), expressed on various immune cells. LIGHT–HVEM signaling has been shown to promote pro-inflammatory responses, including induction of Th1-type immunity and enhancement of NK and CD8^+^ T-cell activity (203, 204). In contrast, LIGHT–LTβR interactions can induce apoptosis in LTβR-expressing tumor cells, suggesting a mechanism for direct, antigen-independent tumor cell killing (205). Cai et al. demonstrated that LIGHT-armoring enhanced CAR T-cell proliferation and cytotoxic function *in vitro*, including against tumor cell lines with heterogeneous antigen expression. *In vivo*, LIGHT-armored CAR T-cells showed increased tumor infiltration and improved tumor control in immunodeficient mouse models, with no detectable toxicities observed in immunocompetent mice (202).

## Discussion and future perspectives

CAR T-cells must surmount multiple barriers in order to effectively eradicate tumor cells, likely accounting for the limited clinical efficacy observed thus far in the treatment of solid tumors. Armoring CAR T-cells with a biological payload, such as molecules that enhance effector function, counteract immunosuppression within the TME, or recruit endogenous immune cells to support the anti-tumor response, has shown promise in numerous preclinical studies, often outperforming unarmored CAR T-cells in specific settings. However, the reproducibility and durability of these responses in the clinical setting remain uncertain. To date, no armored CAR T-cell product has progressed beyond early phase clinical trials, and only limited data from completed or ongoing phase I trials are available. Notably, some strategies, such as IL-12, IL-15 and dnTGF-βRII-armoring, have produced toxicity in phase I trials that were not fully anticipated by preclinical data, underscoring one of the major limitations in this field: the lack of a fully predictive preclinical model.

The large majority of CAR T-cell *in vivo* studies in mice thus far have been carried out in immunodeficient mice, predominantly NSG mice which lack T-cells, B-cells, and NK cells (206). While these models enable the study of human CAR T-cells, which may be perceived as a translational advantage, they fail to recapitulate key immune-mediated toxicities seen in the clinical setting. This is particularly problematic for armored CAR T-cells, where cross-species incompatibilities may prevent interaction of the payload with murine immune components. For instance, human IL-12, one of the most extensively studied cytokine payloads, does not cross-react with murine cells (207). Syngeneic immunocompetent mouse models do provide a method of investigating armored CAR T-cell therapy in organisms that have an intact immune system, and eliminate cross-species barriers. However, these models are not invulnerable to uncertainty or criticism either, given the biological differences between mice and humans. Humanized mouse models, or immunodeficient mice reconstituted with a humanized immune system, have been gaining attention, but difficulties remain in navigating the mismatch of the murine and human systems to establish stable models (206). Alternative platforms, such as canine (208), zebrafish (209), and patient-derived tumor organoid models (128, 210), offer additional insights, but are not widely adopted or accessible. As such, mouse models will likely remain the cornerstone of preclinical CAR T-cell evaluation in the near term. It will continue to be necessary to test armored CAR T-cells in multiple preclinical models to assess different aspects of safety and efficacy. Other challenges highlighted previously in this review, including the pleiotropic effects of different armoring payloads and the heterogeneity of solid tumors, also need to be navigated by judicious selection of a range of preclinical assays and models.

Another layer of complexity arises in defining what constitutes a “beneficial” effect of armoring on CAR T-cell phenotype and function. Many studies highlight increased proliferation, reduced expression of exhaustion markers, and enhanced effector molecule release as positive outcomes. Likewise, the enrichment of memory T-cell subsets, particularly central memory or stem cell-like memory subsets, is generally viewed as being beneficial, due to evidence from studies that demonstrated that these T-cells exhibit superior persistence and induce superior anti-tumor immunity compared to effector memory T-cells (211, 212). However, other studies have conversely found that more differentiated or effector memory-like T-cells could potentially have particular benefits for the therapy of solid tumors, as opposed to hematological malignancies (213–215). Ultimately, only clinical trials in human patients will be able to determine whether the effects on T-cell phenotype induced by armoring payloads in preclinical studies translate to durable benefits for patients with cancer.

As armoring strategies for enhancing the potency of CAR T-cells continue to advance, it is of paramount importance that progress is similarly made in developing mechanisms to ensure the safety of these therapies. As illustrated by a number of studies we have discussed, armoring strategies have evolved to become more refined in regulating expression of the payload, which is important for avoiding off-tumor effects. For example, harnessing the intrinsic biological effects of cytokine signaling is attractive as an armoring approach, but constitutive or uncontrolled cytokine signaling can cause severe toxicities. To address this, researchers have developed a range of expression control strategies, each with their strengths and pitfalls, including tethering cytokines to CAR T-cell membranes, targeting cytokines to tumors via tumor-selective peptides, designing chimeric cytokine receptors, and engineering synthetic promoters and gene circuits responsive to specific intracellular or environmental signals, such as the NFAT-activated promoter system and the synNotch receptor system. The NFAT-activated promoter approach has been widely employed as a means to link payload expression to CAR activation, but has also been found to allow ‘leaky’, or non-tumor-restricted, expression of the payload. For example, a first-in-human trial of tumor-infiltrating lymphocytes (TILs) armored with NFAT-inducible IL-12 was terminated early due to severe toxicities secondary to the secreted IL-12, peak serum levels of which were unpredictable and varied widely, including reaching potentially lethal levels (47). This highlights a limitation of inducible expression systems where the stimulus is CAR or TCR activation, rather than a bona fide tumor-specific stimulus. Efforts to reduce the leakiness of the NFAT promoter system have included utilizing affinity-tuned CARs. One group found that the threshold of CAR target antigen density for NFAT activation was inversely correlated with CAR affinity, such that restriction of NFAT activation to antigen-high tumors was more stringent with low-affinity CARs (216). Another recent study aimed to develop a tumor-inducible expression system by screening for endogenous genes that are differentially upregulated by CAR T-cells in the tumor compared to a non-tumor site, and identified *NR4A2* and *RGS16* as the two most promising candidates. Placing transgene expression under control of these two endogenous promoters through a CRISPR knock-in approach resulted in stringent tumor-restricted expression, and CAR T-cells armored in this manner with tumor-restricted IL-12 or IL-2 exhibited greater anti-tumor efficacy (217). Alternative approaches for linking transgene expression to a TME-intrinsic stimulus include hypoxia-induced expression, which exploits the hypoxic microenvironment of solid tumors (151, 218–220), and synNotch receptor systems, which utilize TAAs or other targets within the TME to induce transgene expression (75, 76, 133, 221). Nonetheless, many switchable technologies have been shown to be prone to leakiness which can result in off-tumor effects. Therefore, tailoring of different armored CAR T-cell therapies not just to tumor types, but to carefully profiled patient and disease-specific characteristics, is likely to become more important as a personalized approach to maximize safety and efficacy.

Finally, apart from engineering inducible expression systems to optimize tumor-restricted expression of the armoring payload, other strategies for improving the safety of armored CAR T-cells have been explored. One approach is to incorporate a module that blocks IL-1 or IL-6 signaling, key mediators of CRS, as the armoring payload itself (222). To avoid systemic immunosuppression, these neutralizing payloads, such as an IL-6 receptor α-blocking antibody, have been regulated using an NFAT promoter-based approach (54), or have been engineered as a chimeric cytokine receptor composed of the extracellular domains of the IL-6 receptor and the intracellular domains of a mutated IL-7 receptor, thereby combining IL-6 sequestration with constitutive IL-7 signaling (223). An alternative approach to alleviating toxicity is to incorporate suicide genes into CAR T-cells, which act as killing switches that rapidly eliminate the CAR T-cells when activated by the appropriate drug. Examples of suicide genes include iC9, which was previously discussed in the context of IL-15 armoring, as well as antibody-dependent cell-mediated cytotoxicity and complement-dependent cytotoxicity switches, where administration of a mAb such as rituximab selectively eliminates CAR T-cells co-expressing epitopes from the corresponding antigen, in this case CD20 (224, 225). As armoring strategies become more advanced, however, involving more complex transgenes and genome engineering, remote approaches for modulating CAR T-cell function that do not require incorporation of another transgene are also appealing. For example, dasatinib is a tyrosine kinase inhibitor that reversibly inhibits CAR signaling, effectively suppressing CAR T-cell functions but allowing these functions to resume when the dasatinib is discontinued (224, 226, 227). This provides a further advantage compared to suicide switches, which permanently eliminate CAR T-cells, potentially impacting anti-tumor activity. Taken together, the range of safety mechanisms that have been developed thus far each offer different benefits, and the selection of the best approach will depend on the desired characteristics and outcomes for the specific CAR T-cell therapy.

Looking ahead, given the myriad barriers to effective CAR T-cell therapy, strategies to refine and combine armoring payload expression are likely to become ever more advanced. A recent example of a combination approach is the study by Erler et al. which described the generation of a multi-armored allogeneic CAR T-cell engineered using TALEN-mediated genome editing technology to achieve knockout of TGF-βRII and PD-1, and knock-in of activation-induced IL-12 (228). Although a comprehensive review of CAR T-cell engineering strategies was beyond the scope of this review, the field is clearly moving toward increasingly sophisticated designs. Future directions will likely involve combining multiple payloads, broadening the scope of payloads beyond secreted proteins, and leveraging advanced genome engineering and synthetic biology tools to tightly regulate CAR T-cell activity. Furthermore, new insights into TME-driven transcriptional and epigenetic reprogramming will continue to inform the rational selection of payloads for the design of next-generation CAR T-cells.

In summary, while armoring strategies have demonstrated significant promise in preclinical models, translation into effective and safe therapies for solid tumors will require a concerted focus on improving preclinical models, refining expression control and safety mechanisms, and deepening our understanding of CAR T-cell biology in the context of solid tumor immunology. The next decade will likely see the emergence of highly engineered, multi-functional CAR T-cells capable of addressing the complex challenges of solid tumors in the clinical setting.
