# Supplementary material for: Current strategies for armoring chimeric antigen receptor T-cells to overcome barriers of the solid tumor microenvironment
Source: Front Immunol. 2025 Sep 11;16:1643941. doi: 10.3389/fimmu.2025.1643941 (PMC12460333; doi:10.3389/fimmu.2025.1643941)
Supplement: Supplementary file 1 [file DataSheet1.pdf]

Supplementary Table 1. Summary of armoring strategies and preclinical models demonstrating benefit

Armoring strategy	Preclinical models: target antigen, tumor type, type of animal model	References
**Exploiting cytokine signaling**		
IL-15	GD2, human neuroblastoma, immunodeficient mouse	(26)
	GD2, human glioblastoma, immunodeficient mouse	(27)
	Claudin18.2, human gastric cancer, immunodeficient mouse	(28)
	VEGFR2, mouse melanoma, immunocompetent mouse	(29)
	Claudin18.2, mouse pancreatic cancer, immunocompetent mouse<br>Claudin18.2, mouse melanoma, immunocompetent mouse	(30)
	IL-13Rα2, mouse glioma, immunocompetent mouse	(31)
IL-15 + IL-21	GPC3, human hepatocellular carcinoma, immunodeficient mouse	(40)
	GD2, human neuroblastoma, immunodeficient mouse	(41)
IL-12	B7-H3, human osteosarcoma, immunodeficient mouse	(49)
	TAG-72, human ovarian cancer, immunodeficient mouse<br>HER2, human breast cancer, immunodeficient mouse<br>TAG-72, mouse ovarian cancer, immunocompetent mouse	(50)
	CEA (antigen-negative model), human ovarian cancer, immunodeficient mouse	(51)
	STEAP1, mouse prostate cancer, immunocompetent mouse	(52)
	Mesothelin, human pancreatic cancer, immunodeficient mouse	(53)

Armoring strategy	Preclinical models: target antigen, tumor type, type of animal model	References
	HER2, human ovarian cancer, immunodeficient mouse	(54)
	GPC3, human hepatocellular carcinoma, immunodeficient mouse<br>GPC3, mouse liver cancer, immunocompetent mouse	(55)
IL-12: affinity-tuned CAR	EpCAM, human gastric cancer, immunodeficient mouse<br>EpCAM, human thyroid cancer, immunodeficient mouse	(216)
IL-12: endogenous promoter-induced	Lewis Y, human ovarian cancer, immunodeficient mouse<br>HER2, mouse breast cancer, immunocompetent mouse<br>HER2, mouse colon cancer, immunocompetent mouse	(217)
IL-23	GD2, human neuroblastoma, immunodeficient mouse<br>B7-H3, human pancreatic cancer, immunodeficient mouse<br>B7-H3, mouse pancreatic cancer, immunocompetent mouse	(56)
IL-2 + IL-33	TRP1, mouse melanoma, immunocompetent mouse<br>B7-H6, mouse melanoma, immunocompetent mouse<br>Rae1, mouse colon cancer, immunocompetent mouse	(59)
IL-18	$MUC16^{ecto}$, mouse ovarian cancer, immunocompetent mouse	(62)
	CEA, human lung cancer, immunodeficient mouse<br>CEA, mouse pancreatic cancer, immunocompetent mouse<br>CEA, mouse colon cancer, immunocompetent mouse	(63)
	DLL3, human small cell lung cancer, immunodeficient mouse<br>DLL3, mouse small cell lung cancer, immunocompetent mouse	(64)
	ErbB, human breast cancer, immunodeficient mouse<br>ErbB, mouse head and neck squamous cell carcinoma, immunocompetent mouse	(65)

Armoring strategy	Preclinical models: target antigen, tumor type, type of animal model	References
	GD2, human neuroblastoma, immunodeficient mouse	(66)
	EphA2, human Ewing sarcoma, immunodeficient mouse<br>HER2, human osteosarcoma, immunodeficient mouse	(67)
IL-7 receptor	GD2, human neuroblastoma, immunodeficient mouse<br>EphA2, human glioblastoma, immunodeficient mouse	(68)
	B7-H3, human glioblastoma, immunodeficient mouse	(70)
	CD44 and CD133, human glioblastoma, immunodeficient mouse	(71)
Orthogonal IL-2/IL-9 chimeric receptor	Mesothelin, mouse pancreatic cancer, immunocompetent mouse	(73)
IL-2: chimeric cytokine receptor with leucine zippers	B7-H3, human lung cancer, immunodeficient mouse<br>EphA2, human Ewing sarcoma, immunodeficient mouse	(74)
IL-2: synNotch-induced	NY-ESO-1, human melanoma, immunodeficient mouse<br>Mesothelin, mouse pancreatic cancer, immunocompetent mouse	(76)
IL-2: endogenous promoter-induced	Lewis Y, human ovarian cancer, immunodeficient mouse<br>HER2, mouse breast cancer, immunocompetent mouse	(217)
**Combating immune-inhibitory signals**		
Inhibiting TGF-β signaling	PSMA, human prostate cancer, immunodeficient mouse	(79)
	STEAP2, human prostate cancer, immunodeficient mouse	(80)
	Mesothelin, human ovarian cancer, immunodeficient mouse	(81)
	Mesothelin, human ovarian cancer, immunodeficient mouse	(82)
	ROR1, human pancreatic cancer, immunodeficient mouse	(83)

Armoring strategy	Preclinical models: target antigen, tumor type, type of animal model	References
	Claudin18.2, human esophagogastric cancer, immunodeficient mouse<br>Claudin18.2, human pancreatic cancer, immunodeficient mouse	(84)
	EGFR and IL-13Rα2, human glioblastoma, immunodeficient mouse	(85)
	IL-13Rα2, human glioblastoma, immunodeficient mouse<br>IL-13Rα2, mouse glioma, immunocompetent mouse	(91)
	EGFR, human lung cancer, immunodeficient mouse	(92)
	HER2, human cervical cancer, immunodeficient mouse	(93)
	Mesothelin, human lung cancer, immunodeficient mouse<br>Mesothelin, human pancreatic cancer, immunodeficient mouse	(94)
Inverted TGF-β/IL-15 receptor	EGFR, human colon cancer, immunodeficient mouse	(86)
Inhibiting PD-1/PD-L1 immune checkpoint	Mesothelin, human lung cancer, immunodeficient mouse	(98)
	$MUC16^{ecto}$, human ovarian cancer, immunodeficient mouse<br>$MUC16^{ecto}$, mouse ovarian cancer, immunocompetent mouse	(99)
	CD133, human hepatocellular carcinoma, immunodeficient mouse	(100)
	ROR1, human breast cancer, immunodeficient mouse	(101)
	Mesothelin and PD-L1, human pancreatic cancer, immunodeficient mouse	(102)
	Carbonic anhydrase IX, human renal cell carcinoma, humanized mouse	(103)

**Armoring strategy**	**Preclinical models: target antigen, tumor type, type of animal model**	**References**
Blocking PD-1 and TREM2	CEA, mouse colon cancer, immunocompetent mouse	(105)
Blocking PD-1 and TGF-β	CD19, human prostate cancer, immunodeficient mouse	(107)
Inhibiting CD47 immune checkpoint	Trop2, mouse colon cancer, immunocompetent mouse	(109)
	EGFRvIII, human glioblastoma, immunodeficient mouse	(110)
	PD-L1, mouse melanoma, immunocompetent mouse<br>EIIIB (splice variant of fibronectin), mouse melanoma, immunocompetent mouse	(111)
	HER2, human osteosarcoma, immunodeficient mouse<br>B7-H3, human neuroblastoma, immunodeficient mouse	(112)
Inhibiting adenosine-A_{2A}R checkpoint	CD19, human ovarian cancer, immunodeficient mouse	(117)
	HER2, human lung cancer, immunodeficient mouse<br>GPC3, human hepatocellular carcinoma, immunodeficient mouse	(118)
	Mesothelin, human ovarian cancer, immunodeficient mouse	(119)
	Lewis Y, human ovarian cancer, immunodeficient mouse	(120)
	Mesothelin, human lung cancer, immunodeficient mouse<br>Mesothelin, human pancreatic cancer, immunodeficient mouse	(121)
Targeting CAFs	Mesothelin, human pancreatic cancer, immunodeficient mouse	(128)
Targeting the ECM	GD2, human neuroblastoma, immunodeficient mouse<br>CSPG4, human melanoma, immunodeficient mouse	(130)
	Mesothelin, human gastric cancer, immunodeficient mouse	(131)

Armoring strategy	Preclinical models: target antigen, tumor type, type of animal model	References
	GPC3, human hepatocellular carcinoma, immunodeficient mouse	(132)
	HER2, human cervical cancer, immunodeficient mouse	(133)
**Modulating metabolic pathways**		
GLUT1	CD19, human renal cell carcinoma, immunodeficient mouse<br>IL-13Rα2, human glioblastoma, immunodeficient mouse	(140)
GLUT3	CD276, human pancreatic cancer, immunodeficient mouse<br>CD276, human esophageal cancer, immunodeficient mouse<br>Mesothelin, human lung cancer, immunodeficient mouse<br>CD276, mouse lung cancer, immunocompetent mouse	(141)
TAGLN2	FSH receptor, mouse ovarian cancer, immunocompetent mouse	(142)
PRODH2	HER2, human breast cancer, immunodeficient mouse	(145)
IL-10	CD19, human pancreatic tumor, immunodeficient mouse<br>HER2, mouse colon cancer, immunocompetent mouse<br>TRP-1, mouse melanoma, immunocompetent mouse<br>EGFRvIII, mouse breast cancer, immunocompetent mouse	(146)
PGC-1α	EGFR, human lung cancer, immunodeficient mouse	(149)
**Overcoming antigen heterogeneity and antigen escape**		
Bispecific immune cell engagers	EGFRvIII/EGFR, human glioblastoma, immunodeficient mouse	(153)
	Muc16/WT1, human ovarian cancer, immunodeficient mouse	(155)

Armoring strategy	Preclinical models: target antigen, tumor type, type of animal model	References
	GPC2/GD2, human neuroblastoma, immunodeficient mouse	(157)
Targeting PRRs: flagellin	CD19, mouse melanoma, immunocompetent mouse<br>CD19, mouse colon cancer, immunocompetent mouse	(160)
Targeting PRRs: neutrophil-activating protein	CD19, mouse neuroblastoma, immunocompetent mouse<br>CD19, mouse pancreatic cancer, immunocompetent mouse<br>PSCA, mouse colon cancer, immunocompetent mouse	(163)
Targeting PRRs: RN7SL1	CD19, mouse melanoma, immunocompetent mouse<br>CD19, mouse lung cancer, immunocompetent mouse<br>Mesothelin, mouse lung cancer, immunocompetent mouse	(165)
Flt3L + poly(I:C)	HER2, mouse breast cancer, immunocompetent mouse<br>HER2, mouse colon cancer, immunocompetent mouse	(168)
Reducing trogocytosis	Mesothelin, human mesothelioma, immunodeficient mouse<br>CD19, mouse melanoma, immunodeficient mouse	(172)
**Improving homing to tumors**		
CXCR1/CXCR2	CD70, human glioblastoma, immunodeficient mouse<br>CD70, human ovarian cancer, immunodeficient mouse<br>CD70, human pancreatic cancer, immunodeficient mouse	(176)
	B7-H3, human rhabdomyosarcoma, immunodeficient mouse	(177)
	avβ6 integrin, human pancreatic cancer, immunodeficient mouse<br>avβ6 integrin, human ovarian cancer, immunodeficient mouse	(178)
	B7-H3, human osteosarcoma, immunodeficient mouse	(179)
	GPC3, human liver cancer, immunodeficient mouse	(180)

Armoring strategy	Preclinical models: target antigen, tumor type, type of animal model	References
CXCR2 + IL-15/IL-18	EGFRvIII, mouse breast cancer, immunocompetent mouse	(181)
CXCR6	B7-H3, human osteosarcoma, immunodeficient mouse	(179)
	Mesothelin, human pancreatic cancer, immunodeficient mouse<br>EpCAM, mouse pancreatic cancer, immunocompetent mouse	(182)
CCR2b	Mesothelin, human mesothelioma, immunodeficient mouse	(183)
	B7-H3, human lung cancer, immunodeficient mouse	(184)
	Mesothelin, human lung cancer, immunodeficient mouse	(185)
CCR2b + IL-7	GD2, human neuroblastoma, immunodeficient mouse	(187)
CXCR5	EGFR, human lung cancer, immunodeficient mouse	(186)
CXCR5 + IL-7	NKG2D, human osteosarcoma, immunodeficient mouse	(188)
CCR5 + IL-12	Mesothelin, human esophageal cancer, immunodeficient mouse	(189)
CCR8 + dnTGF-βRII	Mesothelin, human pancreatic cancer, immunodeficient mouse<br>EpCAM, mouse pancreatic cancer, immunocompetent mouse	(190)
CCL19 + IL-7	CD20, mouse lung cancer, immunocompetent mouse<br>Mesothelin, mouse pancreatic cancer, immunocompetent mouse	(193)
	GM2, human small cell lung cancer, immunodeficient mouse	(194)
	GPC3, human liver cancer, humanized mouse	(195)
	EGFRvIII, human glioblastoma, immunodeficient mouse<br>HER2, human pancreatic cancer, immunodeficient mouse	(196)

Armoring strategy	Preclinical models: target antigen, tumor type, type of animal model	References
CCL21 + IL-7	Claudin18.2, mouse pancreatic cancer, immunocompetent mouse<br>Claudin18.2, mouse liver cancer, immunocompetent mouse<br>Claudin18.2, mouse breast cancer, immunocompetent mouse	(197)
LIGHT	PSMA, human prostate cancer, immunodeficient mouse	(201)
	Mesothelin, human pancreatic cancer, immunodeficient mouse	(202)
**Multi-armoring**		
PD-1 knockout, TGF-βRII knockout, IL-12 knock-in	MUC1, human breast cancer, immunodeficient mouse	(228)
